# Vividness of visual imagery questionnaire scores and their relationship to visual short-term memory performance

**DOI:** 10.1016/j.cortex.2021.10.011

**Published:** 2022-01

**Authors:** Younes Adam Tabi, Maria Raquel Maio, Bahaaeddin Attaallah, Shannon Dickson, Daniel Drew, Mohamad Imran Idris, Annika Kienast, Verena Klar, Lisa Nobis, Olivia Plant, Youssuf Saleh, Timothy Ravinder Sandhu, Ellie Slavkova, Sofia Toniolo, Nahid Zokaei, Sanjay G. Manohar, Masud Husain

**Affiliations:** a Nuffield Department of Clinical Neurosciences, John Radcliffe Hospital, Oxford, UK; b Department of Experimental Psychology, University of Oxford, Oxford, UK; c Department of Psychiatry, University of Oxford, Oxford, UK; d MRC Cognition and Brain Sciences Unit, University of Cambridge, Cambridge, UK; e Department of Psychology, University of Cambridge, Cambridge, UK; f NIHR Oxford Biomedical Research Centre, Oxford, UK; g Wellcome Centre for Integrative Neuroimaging, Oxford, UK

**Keywords:** Visual imagery, Working memory, Hippocampus, Alzheimer's disease, Parkinson's disease

## Abstract

Mechanisms underlying visual imagery, the ability to create vivid mental representations of a scene in the absence of sensory input, remain to be fully understood. Some previous studies have proposed that visual imagery might be related to visual short-term memory (STM), with a common mechanism involving retention of visual information over short periods of time. Other observations have shown a strong relationship between visual imagery and functional activity in the hippocampus and primary visual cortex, both regions also associated with visual STM. Here we examined the relationship of visual imagery to STM and hippocampal and primary visual cortex volumes, first in a large sample of healthy people across a large age range (*N =* 229 behavioural data; *N =* 56 MRI data in older participants) and then in patients with Alzheimer's disease and Parkinson's disease (*N =* 19 in each group compared to 19 age-matched healthy controls).

We used a variant of the “What was where?” visual object-location binding task to assess the quality of remembered information over short delays. In healthy people, no evidence of a relationship between the vividness of visual imagery and any visual STM performance parameter was found. However, there was a significant positive correlation between visual imagery and the volumes of the hippocampus and primary visual cortex. Although visual STM performance was significantly impaired in patients with Alzheimer's disease, their vividness of visual imagery scores were comparable to those of age-matched elderly controls and patients with Parkinson's disease. Despite hippocampal volumes also being reduced in Alzheimer's patients, there appeared to be no impact on their self-reported visual imagery. In conclusion, visual imagery was not significantly related to visual STM performance, either in healthy controls or Alzheimer's or Parkinson's disease but it was related to hippocampal and visual cortex volume in healthy people.

## 1 Introduction

Brain mechanisms underlying visual imagery, the ability to create a vivid visual mental representation of a scene in the absence of sensory input, have long been debated, with much interest focused on the relationship between perception and imagery (Bosch & Gerven, 2019; Kosslyn, 1980; Pylyshyn, 1973). Such considerations have led to a large body of work that has related both the functional activity and structural anatomy of primary visual cortex (V1) to imagery across many different studies (for a review see Pearson, 2019). For example, a meta-analysis of functional imaging studies comprising 464 participants showed V1 to be functionally involved in visual imagery (Winlove et al., 2018).

On the other hand, recent research concluded that visual imagery and visual perception might not share the same neuroanatomical correlates. This conclusion is based on the fact that patients with damage to the left temporal lobe, but fully intact primary visual cortex, were found to be affected in their visual imagery (Moro, Berlucchi, Lerch, Tomaiuolo, & Aglioti, 2008). A patient suffering from lesions to extrastriate visual areas was, however, unimpaired in their ability to revisualize vividly from memory while they were significantly impaired in their visual perceptual abilities (Bartolomeo et al., 1998). Such single cases are further backed by imaging studies that linked the fusiform gyrus but not the visual cortex to visual imagery (Thorudottir et al., 2020, for a review: Spagna, Hajhajate, Liu, & Bartolomeo, 2021). In his review, Bartolomeo (2002) further suggested that damage to the occipital cortex does not necessarily lead to an impairment of visual imagery. However, brain-damage involving higher-visual areas seems related to a loss of the ability to vividly imagine visual contents. Damage to the left fusiform gyrus, for example, induced a deficit in construction of visual images (Bartolomeo, Hajhajate, Liu, & Spagna, 2020). Thus, Bartolomeo and colleagues concluded that the fusiform gyrus might be the essential link “between sensory information coming from the occipital cortex and semantic processing in the anterior temporal lobe”. Further, cases of patients with brain damage involving visual imagery deficit with anatomical data seem to consistently involve the temporal lobe whereas damage to the visual cortex does not necessitate such impact on visual imagery. In a meta-analysis on fMRI data, Spagna et al. (2021) showed the left fusiform gyrus but not primary visual cortex activity to be related to visual imagery. In particular, visual mental imagery did not increase V1 BOLD response (Spagna et al., 2021). Bergmann and colleagues rather find a negative correlation between V1 surface area and sensory imagery strength (Bergmann, Genç, Kohler, Singer, & Pearson, 2016) and Fulford and colleages’ fMRI study and literature review showed the vividness of imagery not only to be negatively correlated to activation in V1 but to be positively correlated to activation in the fusiform gyrus.

A different line of work has suggested that there might also be a link between visual short-term memory (STM) and the vividness of visual imagery (Keogh & Pearson, 2011). In their study, Keogh and Pearson show that volunteers with good imagery also performed well in a two-forced choice memory task in which they were to remember orientations of Gabor patches over short periods of time. Moreover, performance of individuals with strong imagery was affected by a purely perceptual manipulation (background luminance). In a follow-up study, Keogh and Pearson show that good visual imagers have significantly higher STM capacities. This benefit, however, is disrupted in the presence of luminance (Keogh & Pearson, 2014). These findings support early works from Kosslyn (1980) suggesting the key role in imagery of a visual buffer from which people can decode task relevant information to support recall over short periods of time. Further evidence for this theory has been provided through recent fMRI studies. For example, Harrison and Tong showed that it is possible to decode orientation information from the visual cortex over the delay period in a STM task in which participants had to report orientations (Harrison & Tong, 2009). Thus, task-relevant information was retained in early visual areas in the absence of the input stimulus until a response could be given following a delay. Albers and colleagues were even able to decode activity patterns from the early visual cortex when participants were to mentally rotate a grid with the decoded information resembling activity induced by bottom-up visual stimuli (Albers, Kok, Toni, Dijkerman, & De Lange, 2013). In a mental rotation paradigm, Logie and colleagues showed high-vividness imagers to have significantly more cognitive activation during the task than low-imagers. They concluded that high-vividness imagers were sufficiently capable of manipulating visual representations in the absence of appropriate stimuli using the same areas that would be used to perceive an existing stimulus (Logie, Pernet, Buonocore, & Sala, 2011).

Contrastingly, Bona and colleagues showed a dissociation between the perceived vividness of STM content and its vivid perception. The authors presented participants with a memory cue followed by a probe stimulus. Using a forced-choice response, participants were to report the orientation of the probe in relation to the previously seen cue, i.e., whether it was tilted left or right. Afterwards, participants rated the perceived vividness of the previously presented stimulus. In half of the trials, a masked distractor followed the cue. In these trials, participants were also to rate the perceived visibility of the distractor. Only when distractors were very different from the cue did they impair memory performance independent of the degree to which the distractor was perceived as visible. The vividness of the cue was impaired by distractors of all orientations as long as the latter were perceived as invisible. The authors concluded that subjective perception of memory content, such as vividness, is dissociable from objectively remembered features. Further, Cooper and colleagues very recently showed that the number of details remembered is not necessarily correlated to subjectively perceived vividness of memoranda (Cooper, Kensinger, & Ritchey, 2019). Their study shows that someone who does not remember many details might still remember these vividly and vice versa.

In addition to the visual cortex, there is another structure in the brain that has often been linked to both visual STM and visual imagery: the hippocampus. For example, Hassabis and colleagues demonstrated that the mental construction of a new fictitious scene involves increased activity in the hippocampus (Hassabis, Kumaran, Vann, & Maguire, 2007). Other authors have shown that such hippocampal activation is related to the boundaries of a new scene of which participants were asked to imagine being part of (Bird, Capponi, King, Doeller, & Burgess, 2010). In the visual STM literature too, many previous studies have reported a positive correlation of memory performance with human hippocampal activity or lesions of the hippocampus (i.e., Axmacher et al., 2007; Pertzov et al., 2013).

While some of these findings support the concept of a direct link between visual STM and visual imagery, others have cast some doubt on this proposal. In Keogh and Pearson's (2011) aforementioned study, people with low imagery ability could also perform above chance and the authors suggested this might be explained by these individuals using a different strategy. Further, in an individual with very poor or no visual imagery, a condition called *aphantasia,* Jacobs and colleagues found that STM performance is only impaired in the most demanding task conditions, but not in general (Jacobs, Schwarzkopf, & Silvanto, 2018).

Possible shortcomings of these previous investigations might be the use of binary report (correct/incorrect) measures of memory rather than continuous, analogue STM measures (Pertzov et al., 2013) which might be more sensitive. In addition, low participant numbers in previous investigations might have reduced power to detect effects. In the present study, we used a delayed reproduction task which permits assessment of the precision of recall (Ma, Husain, & Bays, 2014). They index the quality of representations in memory by requiring the participant at test to reproduce features of an item they had seen previously, using a continuous dimension. For example, they might be asked to reproduce the exact spatial location of an item (in x- and y-coordinates) instead of simply giving them a choice of two locations.

In the current investigation, participants were presented on a touchscreen with a set of fractal stimuli, the appearance and screen locations of which they were asked to remember. At test, in the first part of their response they first performed a traditional, binary two-forced-choice task: they were presented with two items (one which they had seen previously, the target, and a novel distractor) and asked to select the target. Next, they were asked to report the original location of the target by dragging it on the touchscreen to its exact remembered location, making it possible to measure their precision of memory recall on a continuous, analogue scale (Liang et al., 2016).

In a first study, a large sample (*N =* 229) healthy participants aged between 26 and 81 years were tested on this STM task in addition to performing the vividness of visual imagery questionnaire (**VVIQ**, Marks, 1995), a widely established measure in the visual imagery research which proved also easy to administer to older individuals and people with neurological disorders. In addition, 56 of these participants underwent a structural MRI brain scan. We sought to test two hypotheses:

• Self-reported visual imagery ability is related to visual STM
• Self-reported visual imagery is related to the volume of the hippocampus, primary visual cortex and fusiform gyrus, but not to control regions like the primary motor cortex (cortical control) or the amygdala (subcortical control).

If the first hypothesis holds true and there is a positive correlation between visual imagery and STM in heathy individuals, we might also expect a patient group that typically has an impairment in memory performance to suffer a decrease in vividness of visual imagery. This is especially true if the vividness of visual imagery is in fact a valid strategy in STM recall as proposed by Keogh and Pearson (2011). Further, if neurodegeneration is pronounced in the hippocampus, a decrease of visual imagery would be expected if the second hypothesis were correct.

One group of neurological patients that presents with clinical memory impairment and atrophy of the hippocampus (decrease of hippocampal volume) is classically people with Alzheimer's disease. Thus, in a second study, we also tested Alzheimer's disease patients and compared them to an equal number of healthy controls of similar age. Because a reduction of visual imagery might be a general symptom of neurological disease and not necessarily associated with a decrease in memory *per se*, a second group of patients suffering from a different neurodegenerative disorder, Parkinson's disease, was also recruited to act as control for the general effects of neurodegeneration.

Research on visual imagery in Alzheimer's disease and Parkinson's disease is relatively sparse. In Alzheimer's disease, some studies report a decrease of visual imagery when patients relive their memories (El Haj, Kapogiannis, & Antoine, 2016; El Haj, Moustafa, et al., 2019), but others have found no difference in VVIQ scores between Alzheimer's disease patients and controls (El Haj, Badcock, et al., 2019). In terms of performance on a delayed STM reproduction task, as the one used in our study, both familial and sporadic Alzheimer's disease cases show deficits (Liang et al., 2016; Zokaei, Sillence, et al., 2020), specifically with respect to misbinding – the ability to hold intact the identity of the fractal and its location, on this task. Further, this specific type of error has been linked to dysfunction of the hippocampus which is known to be involved in feature-object binding (Liang et al., 2016; Pertzov et al., 2013). Thus, if the same regions in the hippocampus are involved in STM and visual imagery, a decrease in VVIQ scores might be expected in Alzheimer's disease patients.

While one study reported that Parkinson's disease patients score significantly lower on the VVIQ compared to elderly controls (Lo Monaco et al., 2018), another has suggested that visual imagery remains unaffected in Parkinson's disease (Scarpina et al., 2019). On delayed reproduction STM tasks, Parkinson's disease patients showed significantly more random responses, rather than misbinding, compared to healthy controls (Zokaei, Heyes, Gorgoraptis, Budhdeo, & Husain, 2015, pp. 319–329; Zokaei, Sillence, et al., 2020). Though Parkinson's disease is not initially linked to a decrease of hippocampal volume, there is some recent evidence for a hippocampal subfield decrease in this group too (Xu et al., 2020). In consequence, for our second study, we further extended the hypotheses of the first experiment to investigate if these applied also to pathologies associated with STM deficits or hippocampal atrophy.

## 2 General methods

We report how we determined our sample size, all data exclusions (if any), all inclusion/exclusion criteria, whether inclusion/exclusion criteria were established prior to data analysis, all manipulations, and all measures in the study.

### 2.1 Short-term memory task

**Fig. 1:**
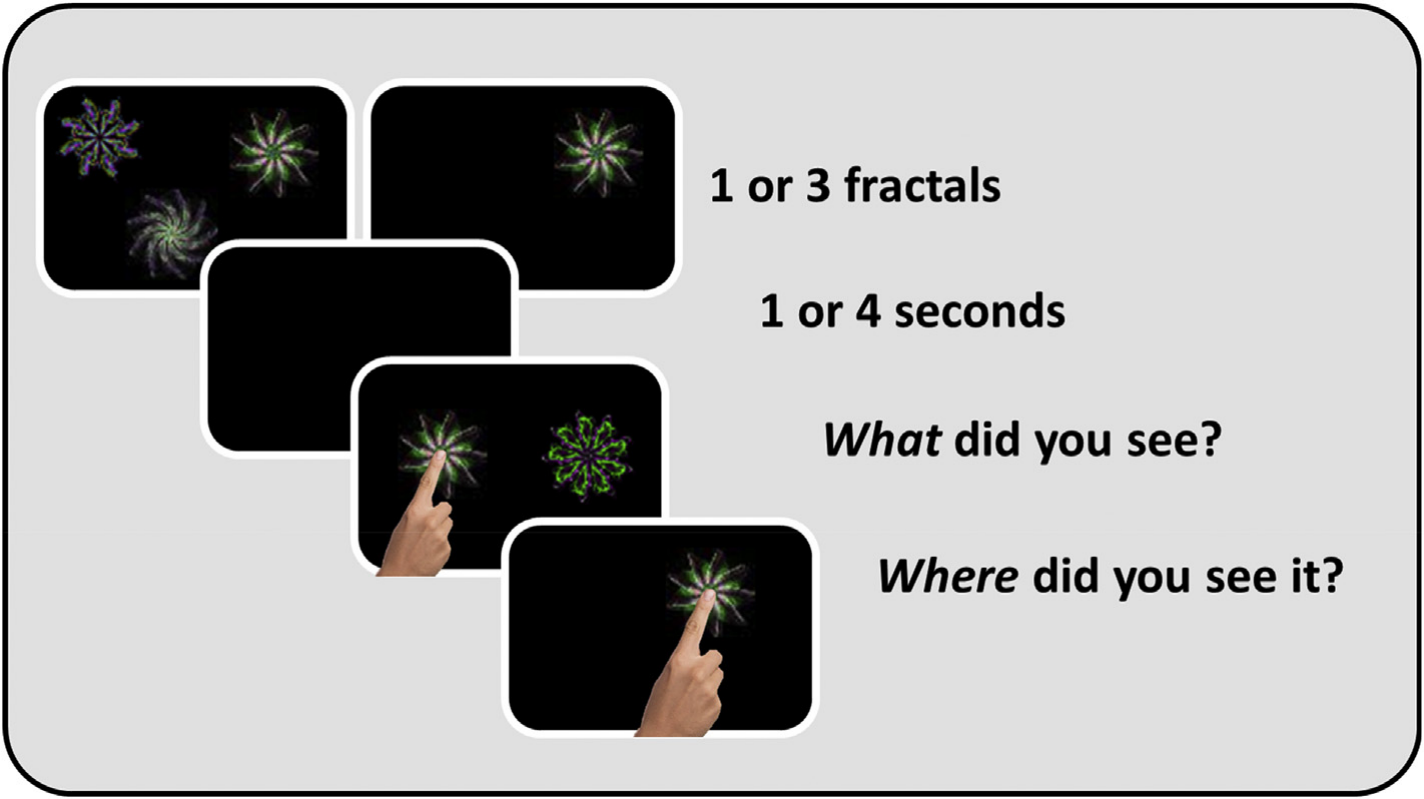
**Task Schematic of the short-term memory task**. Participants were presented with one or three fractals for a period of 1 ssec per fractal. After a blank interval of one or 4 sec, one of the fractals in the original display reappeared together with a distractor fractal. Participants first had to select the fractal which they thought had been presented previously (Identification accuracy) and drag it to where they recalled it had previously appeared (Localisation performance).

A variant of a “What was where?” object-location visual STM task (Fig. 1) was used to measure STM (Pertzov et al., 2013). Volunteers sat at a viewing distance of approximately 30 cm. The task was performed on a touch-sensitive screen (iPad version 9.3.5 (13G36), model MGTX2B/A) with a 1536 × 2048-pixel matrix (Zokaei, Grogan, et al., 2020). Stimuli were presented on a black background and were drawn from a library of foils, randomly selected without repetitions for every trial.

Participants were presented with either one or three random fractal shapes. If one fractal was presented, it appeared for 1 sec; if three fractals were presented, they were displayed for a total of 3 sec. This meant that there was a mean duration for encoding of 1 sec per item. After a delay of one or 4 sec, people were asked to select the shape they had previously seen from two shapes presented at the centre of the screen, one of which was a never-seen, novel foil. In order to select the correct shape, they were asked to touch it. This provided a discrete measure of ***Identification Accuracy***. Then they were requested to drag the selected item across the tablet screen to its remembered location. Hence, participants were not only required to simply remember the appearance of the fractals but to also remember and retrieve information on where they were presented on the tablet screen. This allowed measurement of ***Localisation Performance*** on an analogue scale: the distance between the true location where the fractal had appeared and the location remembered by the participant.

As soon as the person stopped dragging the chosen shape, a “Done” button appeared in the bottom centre of the screen. This disappeared if participants started dragging the shape again and then reappeared upon release of the shape. The final position was confirmed by them pressing the ‘Done’ button. To continue with the next trial, they simply pressed a “Next” button.

The shapes were randomly drawn from a set without repetitions and presented on a black background. Overall, there were three trials with on-screen instructions, four practice trials for each of the conditions (one *vs* three shapes, one *vs* 4 sec delay). After this introduction, volunteers performed a total of 120 trials over three blocks, leading to a total of 30 trials per condition that were equally distributed across blocks. 154 of the elderly controls performed a shorter version of 87 trials only (7 introductory trials, followed by 20 trials per condition). Design of the task allowed to directly extract the following parameters:

• **Identification Accuracy:** Trials in which the correct shape was identified correctly out of two divided by the total number of trials of each condition. Trials in which participants did not identify the correct item were excluded from further analysis.
• **Reaction Time:** Time taken until this choice was made.
• **Localisation Performance:** Distance between the response and the original target's location.

Two types of error, namely **Misbinding** and **Guessing**, in the Localisation Performance were defined based on the types of error in the mixture model of continuous response errors (Bays & Husain, 2008). Misbinding reflects the probability of a participant correctly remembering the appearances and locations of the shapes at test but to *misbind* these locations and appearances resulting in the report of another shape's location for a correctly identified shape. Guessing indicates a random response which volunteers are likely to give when they have forgotten any information on the target whatsoever.

For each trial, the distances between the response and i) the target, ii) the closest non-target and iii) another random trial's non-target were compared with one another. If the distance to the target was closest, this was counted as a target response. If the distance to the non-target was closest, this was counted as a misbinding and finally if the distance to the random trial's non-target was closest, this was counted as a guessing. For each trial, this procedure was repeated 5000 times with a random trial's non-target each time. Thus, probabilities for each trial for misbinding and guessing could be calculated.

### 2.2 Vividness of visual imagery

All participants were tested on the vividness of visual imagery questionnaire which is a long established metric in the field (**VVIQ**; Marks, 1995). The questionnaire consists of 16 questions on which participants score between 1 and 5 according to how vividly they imagine a familiar person, a shop, the sky or a countryside scene, leading to scores between 16 and 80.

### 2.3 MRI analysis

A 3T Siemens Magnetom Verio syngo scanner was used to acquire T1-weighted volumetric images through a magnetisation prepared rapid gradient echo protocol (MPRAGE) in sagittal orientation (TR = 2000 msec, TE = 1.94 msec, TI = 880 msec, Flip angle = 8°, FOV read = 256 mm, Voxel size = 1.0 × 1.0 × 1.0 mm). The same machine was used to record T2-FLAIR images.

FSL FIRST (Patenaude, Smith, Kennedy, & Jenkinson, 2011) was used to generate hippocampal and amygdala volume for both hemispheres from the T1 anatomical images. These volumes were corrected for age and total intracranial volume which was calculated using FSL SIENAX.

Grey matter volume of BA4, V1 and fusiform gyrus were calculated through the standard Freesurfer pipeline (cortical reconstruction and volumetric segmentation, documented and freely available for download online; http://surfer.nmr.mgh.harvard.edu/) and also corrected for age and total intracranial volume. In addition, subfields of the hippocampus were decomposed using the software's subfield pipeline. These analyses used T1 and T2 image inputs.

### 2.4 Plotting

Result plots were created using Morel's software *Grammar of graphics plotting in Matlab* (Morel, 2018)*.*

### 2.5 Availability of data

All data is provided here: https://osf.io/q37vn/in a format readily readable by a statistics software like JASP. No part of the study procedures or analyses was pre-registered prior to the research being conducted. Legal restrictions that are beyond our control prevent us from publicly archiving the memory task and analysis scripts used in this research. Specifically, for commercial use these can be obtained through licensing agreement with Oxford Innovations Ltd. These digital materials will, however, be shared freely on request with research groups and non-profit making organisations provided they are not shared with commercial parties or used for profit.

### 2.6 Study 1: short-term memory and visual imagery in healthy participants

#### 2.6.1 Participants

229 healthy participants were recruited for testing of visual imagery and visual STM. 56 of these underwent an MRI scan (Demographics in Table 1). 73 of the elderly controls performed the Addenbrooke's Cognitive Examination (ACE-III), an established cognitive screening test which is scored out of 100. All participants gave their informed consent to be involved in the study which was approved by the local ethics committee. Participant numbers in this and the following study were chosen to be at least the number of the previous studies cite in the introduction.

**Table 1:** Demographics for the healthy controls (Controls in the left column also include Elderly Controls with MRIs).

	Controls (N = 229)	Elderly Controls with MRIs (N = 56)
Mean Age	57.70 (SD = 12.68, MIN = 26, MAX = 81)	67.46 (SD = 8.04, MIN = 50, MAX = 80)
Mean ACE	97.15 (SD = 2.65, MIN = 88, MAX = 100)	97.27 (SD = 2.55, MIN = 89, MAX = 100)
Mean VVIQ	61.61 (SD = 13.31, MIN = 16, MAX = 80)	62.27 (SD = 12.41, MIN = 16, MAX = 80)

#### 2.6.2 Results

##### 2.6.2.1 Performance on STM task

**Fig. 2:**
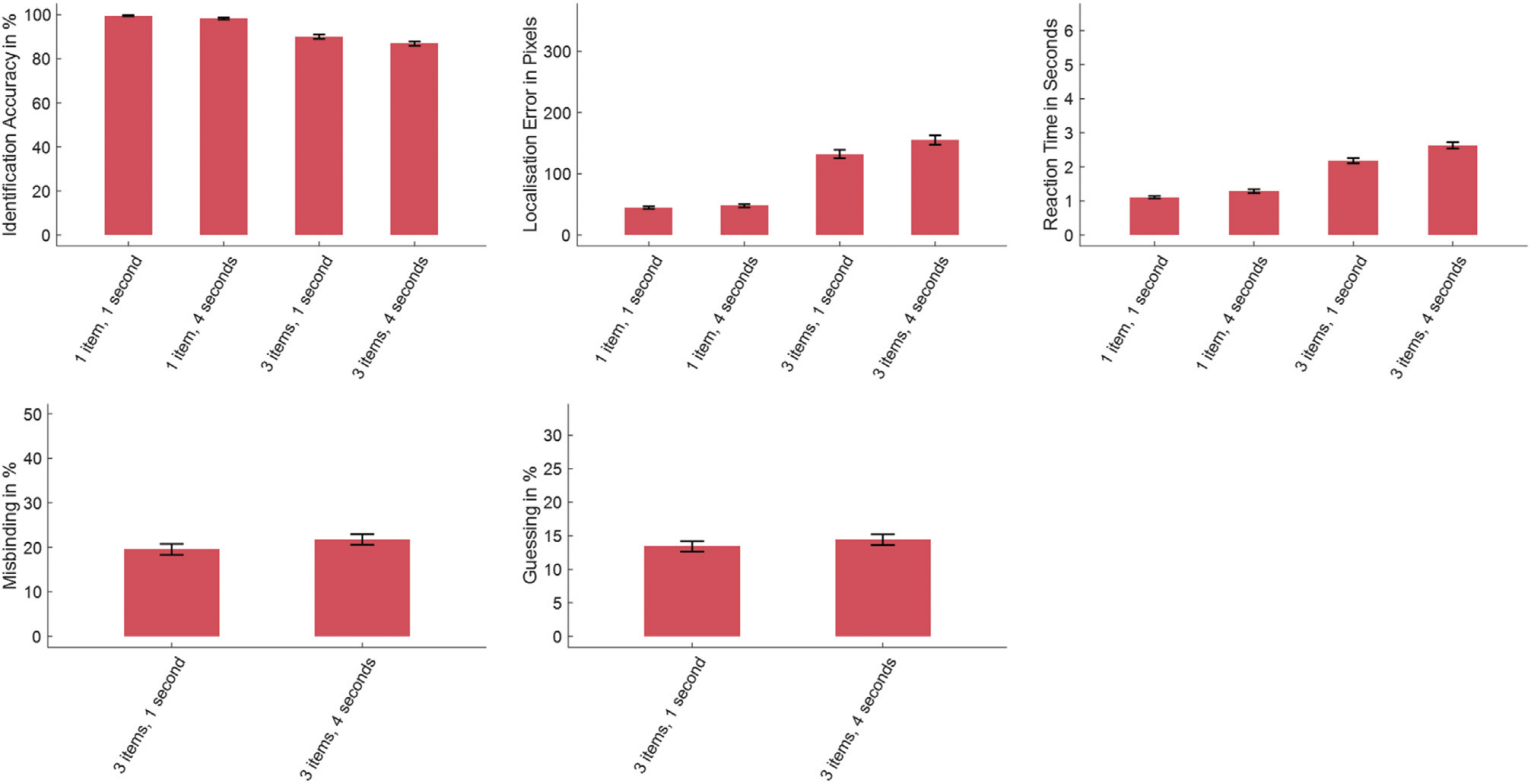
**Results of the short-term memory task in Healthy Controls**. Increase in set size (from one to three) led to higher Localisation Error, lower Identification Accuracy and a longer Reaction Time. An increase of the delay from one to 4 sec worsened all parameters.

As expected, participants performed worse when asked to remember three items compared to one. This was true for both their recall accuracy in identifying the shape (F(1,228) = 721.00, *p* < .001, $\eta_{p}^{2}$ = .760, Fig. 2) and their localisation performance in placing their response as close to the original target's location as possible (F(1,228) = 1154.66, *p* < .001, $\eta_{p}^{2}$ = .835). They also took significantly longer to select the target in three-item trials (F(1,228) = 1183.21, *p* < .001, $\eta_{p}^{2}$ = .838).

With an increase in maintenance delay from one to 4 sec there was a significant decrease in identification accuracy (F(1,228) = 49.65, *p* < .001, $\eta_{p}^{2}$ = .179) and increase in localisation error (F(1,228) = 54.29, *p* < .001, $\eta_{p}^{2}$ = .192). They also showed both significantly increased Misbinding (F(1,228) = 10.95, *p* = .001, $\eta_{p}^{2}$ = .048) and Guessing (F(1,228) = 20.41, *p* < .001, $\eta_{p}^{2}$ = .082) and were also generally slower (F(1,228) = 210.42, *p* < .001, $\eta_{p}^{2}$ = .480).

Details are provided in Supplementary Materials, with interactions of set size and delay in Identification Accuracy, Localisation Performance and Reaction Time decomposed in Supplementary Tables 2, 4 and 8.

##### 2.6.2.2 Visual imagery in relation to STM performance, brain volumes and age

**Fig. 3:**
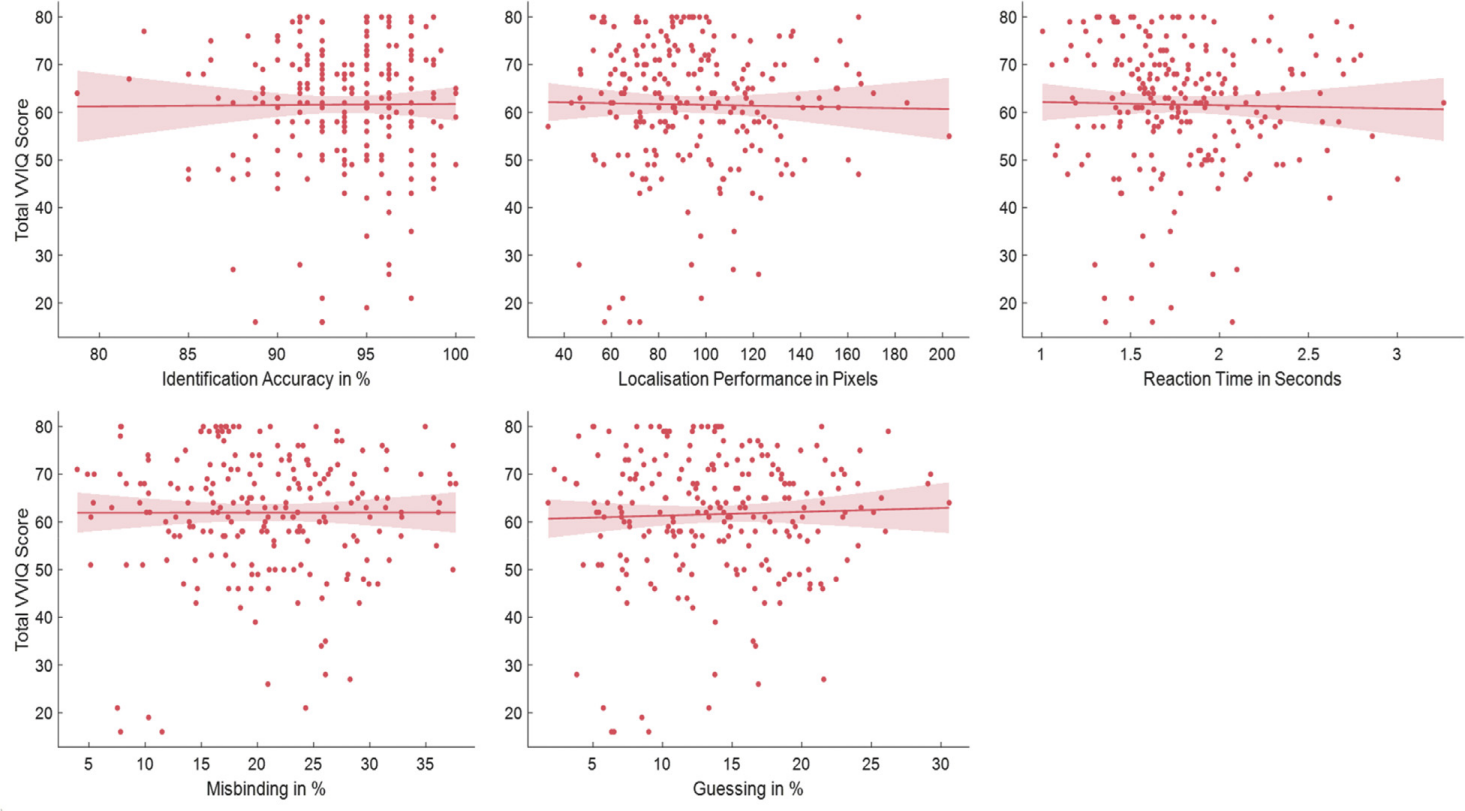
General linear model correlations of VVIQ with Identification Accuracy, Localisation Performance, Reaction Time, Misbinding and Guessing. VVIQ Scores did not correlate with any of the short-term memory task measures, rejecting our first hypothesis and suggesting an independence of visual imagery and visual STM.

There was no significant correlation of VVIQ with Age (r(227) = .09, *p* = .186) or ACE (r(227) = .07, *p* = .560). Nor was there a significant correlation with any of the measures used to index STM performance: identification accuracy, localisation error, Misbinding, Guessing or reaction time (Fig. 3, Supplementary Table 9). Thus, in healthy people, there was no evidence in favour of the hypothesis that visual imagery is related to STM.

**Fig. 4:**
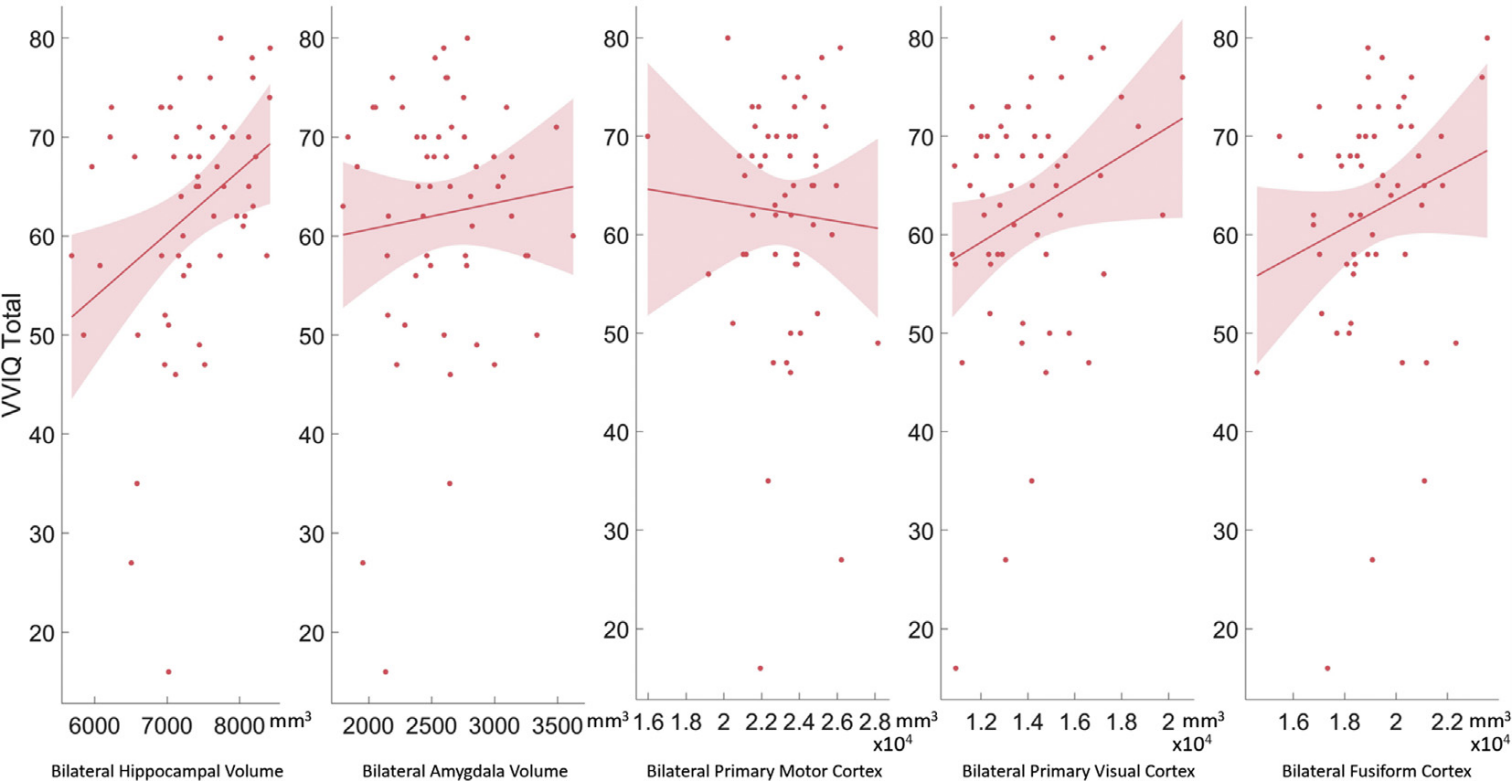
General linear model for correlations of VVIQ and Bilateral Hippocampal Volume, Amygdala Volume, Volume of the Primary Motor Cortex, of the Primary Visual Cortex and of the Fusiform Gyrus. Vividness of Visual Imagery Questionnaire (VVIQ) Scores positively correlated with the volume of the Hippocampus and the Primary Visual Cortex but not with the volume of the Amygdala or the Primary Motor Cortex controls, suggesting an involvement of these two areas in visual imagery and confirming our second hypothesis. There was, however, no correlation of Fusiform gyrus volume and VVIQ.

VVIQ was, however, positively correlated with bilateral hippocampal volumes (r(227) = .35, *p* = .009; left hippocampus: r(227) = .33, *p* = .013; right hippocampus: r(227) = .26, *p* = .050, Supplementary Table 9 for details, Fig. 4) in the 56 Elderly Controls with MRI scans. It was also marginally positively correlated with the bilateral primary visual cortex volume (r(227) = .26, *p* = .050; left primary visual cortex: r(227) = .29, *p* = .032; right primary visual cortex: r(227) = .22, *p* = .107). There was no correlation between VVIQ and the fusiform gyrus volume (left fusiform gyrus: r(227) = .19, *p* = .153; right fusiform gyrus: r(227) = .17, *p* = .201; bilateral fusiform gyrus: r(227) = .20, *p* = .132) or between VVIQ and the primary motor cortex volume (left motor cortex: r(227) = −.01, *p* = .926; right motor cortex: r(227) = −.09, *p* = .533; bilateral motor cortex: r(227) = −.05, *p* = .704).

**Fig. 5:**
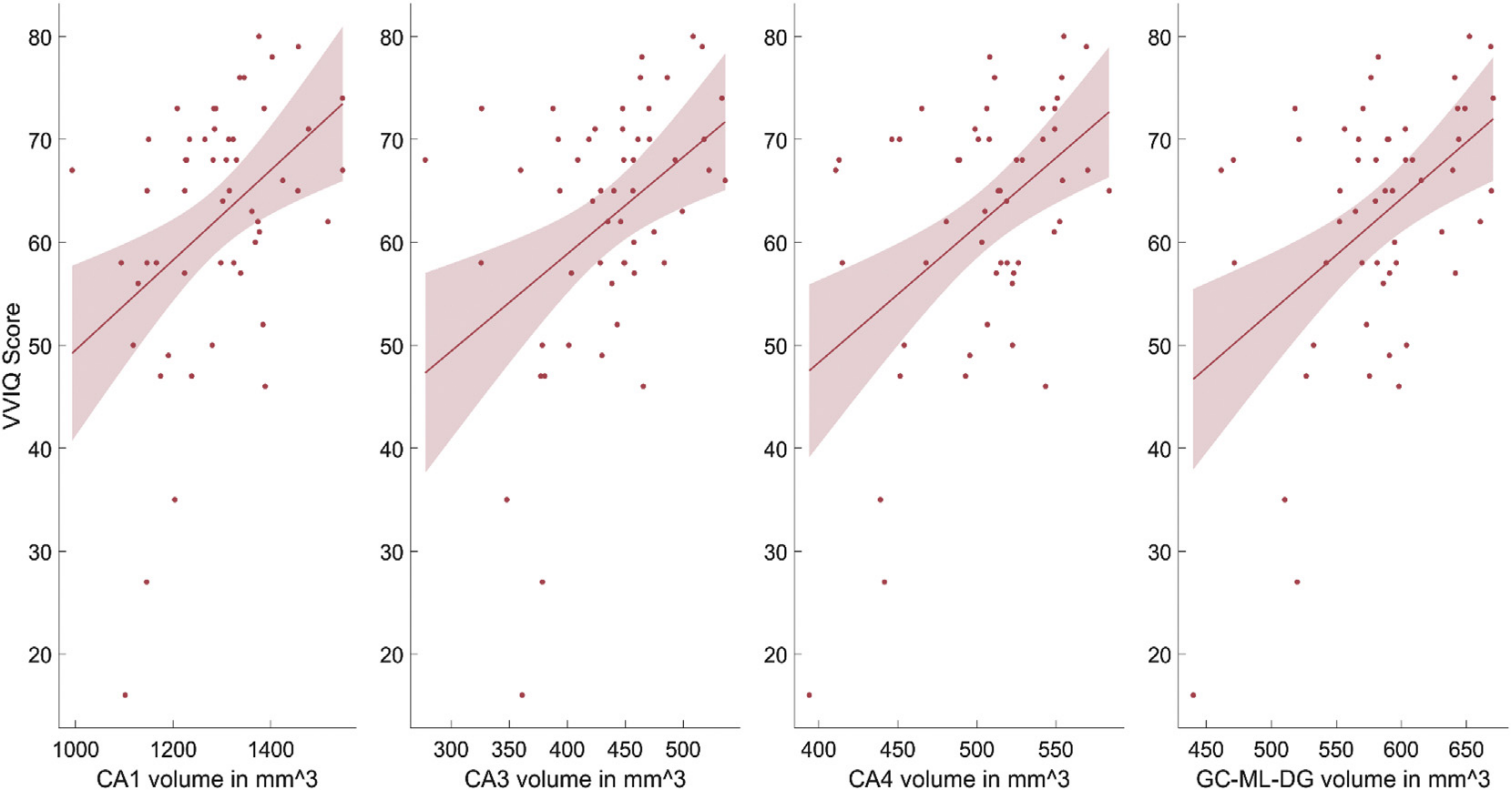
General linear model follow-up for correlations of VVIQ and Bilateral CA1, CA3, CA4 and Granule Cell (GC) and Molecular Layer (ML) of the Dentate Gyrus (DG). The follow-up analysis revealed that visual imagery was in particular correlated with the four subfields presented in the graph.

Following up on the positive correlation with the hippocampus, hippocampal subfield analyses (Supplementary Table 10, Fig. 5) revealed that VVIQ scores were in particular correlated to regions CA1 (r = .42, *p* = .002), CA3 (r = .42, *p* = .002), CA4 (r = .47, *p* < .001) and the Granule Cell (GC) and Molecular Layer (ML) of the Dentate Gyrus (DG, r = .47, *p* < .001). CA1 is known to be reduced in Alzheimer's disease (Khan et al., 2015; Shim et al., 2017), while CA3 has previously been associated with feature binding and auto-association (Rolls, 2018) in STM.

In summary, the results show that while VVIQ is related to hippocampal volume and the primary visual cortex in healthy people, there is no evidence for an association with visual STM. Thus, there is evidence for our second hypothesis, but not the first.

### 2.7 Study 2: short-term memory and imagery in Alzheimer's & Parkinson's disease

#### 2.7.1 Participants

19 Alzheimer's disease patients and 19 Parkinson's disease patients who underwent MRI scanning were recruited for assessment of visual imagery and visual STM. A subset of controls with MRIs from Study 1 was approximately age-matched to these two groups of patients. Demographics for the resulting groups are given in Table 2. All patients performed the Addenbrooke's Cognitive Examination (ACE-III) –a cognitive screening test which is scored out of 100. Patients were recruited from the Cognitive Neurology clinic (via authors MH and SM) and gave their informed consent to be involved in the study which was approved by the local ethics committee.

**Table 2:** Demographics for healthy controls versus Parkinson's disease.

	Elderly controls	Alzheimer's disease patients	Parkinson's disease patients
Mean Age in years	70.37 (SD = 8.32, MIN = 54, MAX = 80)	71.11 (SD = 9.99, MIN = 53, MAX = 89)	69.21 (SD = 7.34, MIN = 52, MAX = 80)
Mean ACE	96.58 (SD = 2.95, MIN = 89, MAX = 100)	74.58∗ (SD = 13.11, MIN = 45, MAX = 96)	95.47 (SD = 2.37, MIN = 92, MAX = 99)
Mean VVIQ	61.16 (SD = 9.21, MIN = 46, MAX = 76)	63.26 (SD = 11.25, MIN = 36, MAX = 80)	69.32∗ (SD = 10.45, MIN = 45, MAX = 80)
Mean Bilateral Hippocampal Volume (mm^3^)	7318.74 (SD = 719.68, MIN = 5961.90, MAX = 8220.91)	6017.07∗ (SD = 733.82, MIN = 4819.91, MAX = 7727.97)	7330.87 (SD = 785.24, MIN = 6040.85, MAX = 8656.33)
Mean Bilateral Amygdala Volume (mm^3^)	2525.51 (SD = 433.75, MIN = 1796.43, MAX = 3489.47)	2172.32∗ (SD = 316.71, MIN = 1729.34, MAX = 2816.08)	2724.49 (SD = 363.41, MIN = 1914.76, MAX = 3243.03)
Mean Bilateral Primary Visual Cortex Volume (mm^3^)	13971.44 (SD = 2750.19, MIN = 10876.31, MAX = 20605.82)	14002.82 (SD = 2072.93, MIN = 10555.07, MAX = 16816.65)	13706.53 (SD = 1978.12, MIN = 9871.03, MAX = 16627.85)
Mean Bilateral Primary Motor Cortex Volume (mm^3^)	23306.06 (SD = 1932.36, MIN = 19188.60, MAX = 28152.79)	23796.44 (SD = 2451.65, MIN = 18934.01, MAX = 28120.25)	23268.03 (SD = 2700.09, MIN = 14176.87, MAX = 27185.24)
Mean Bilateral Fusiform Cortex Volume (mm^3^)	18959.33 (SD = 1931.04, MIN = 14579.38, MAX = 21900.53)	17294.94∗ (SD = 1807.01, MIN = 13874.18, MAX = 20702.69)	18653.62 (SD = 1843.25, MIN = 14493.03, MAX = 22222.333)
Mean Bilateral CA1 Volume (mm^3^)	1259.72 (SD = 121.30, MIN = 1037.25, MAX = 1428.60)	1053.54∗ (SD = 136.94, MIN = 823.12, MAX = 1289.59)	1298.42 (SD = 144.72, MIN = 999.25, MAX = 1521.57)
Mean Bilateral CA3 Volume (mm^3^)	429.15, (SD = 39.95, MIN = 368.96, MAX = 492.79)	318.52∗ (SD = 57.81, MIN = 238.58, MAX = 455.30)	422.29 (SD = 43.95, MIN = 337.26, MAX = 489.78)
Mean Bilateral CA4 Volume (mm^3^)	500.07, (SD = 38.95, MIN = 420.95, MAX = 565.76)	419.10∗ (SD = 41.25, MIN = 352.26, MAX = 508.70)	504.87 (SD = 42.49, MIN = 421.41, MAX = 569.43)
Mean Bilateral GC-ML-DG Volume (mm^3^)	570.31 (SD = 48.74, MIN = 471.83, MAX = 653.23)	483.15∗ (SD = 48.24, MIN = 408.24, MAX = 589.02)	583.62 (SD = 47.39, MIN = 481.96, MAX = 662.63)

Note: Asterisks mark significant differences between patients and controls as defined by independent samples *t*-tests.

#### 2.7.2 Results

##### 2.7.2.1 STM performance

There was a significant main effect of Group for identification recall accuracy (F(2,54) = 18.91, *p* < .001, η_p_^2^ = .412), localisation error (F(2,54) = 14.80, *p* < .001, η_p_^2^ = .354), Misbinding (F(2,54) = 12.30, *p* < .001, η_p_^2^ = .313), Guessing (F(2,54) = 21.58, *p* < .001, η_p_^2^ = .444) and in the time taken to identify the target (F(2,54) = 15.77, *p* < .001, η_p_^2^ = .369). As expected, Holm-corrected post-hoc *t*-tests show that Alzheimer's disease patients identified significantly fewer items correctly than controls (t = 5.25, *p* < .001) and Parkinson's disease patients (t = 4.93, *p* < .001). They also had a significantly greater localisation error than both of these groups (t = 4.46, *p* < .001; t = 4.93, *p* < .001), driven by higher Misbinding (t = 4.23, *p* < .001; t = 4.36, *p* < .001) and Guessing (t = 5.38, *p* < .001; t = 5.96; *p* < .001) in Alzheimer's disease compared to controls and Parkinson's disease patients.

Thus, Alzheimer's disease patients were more likely to either move the target to a non-target's location or to forget information about the locations whatsoever and make a random guess. Alzheimer's disease patients were also significantly slower than controls and Parkinson's disease patients (t = 5.07, *p* < .001; t = 4.62, *p* < .001). Parkinson's disease patients did not differ significantly in their performance compared to controls in any of the measures. That is, they were generally not impaired in their STM performance in comparison to controls.

**Fig. 6:**
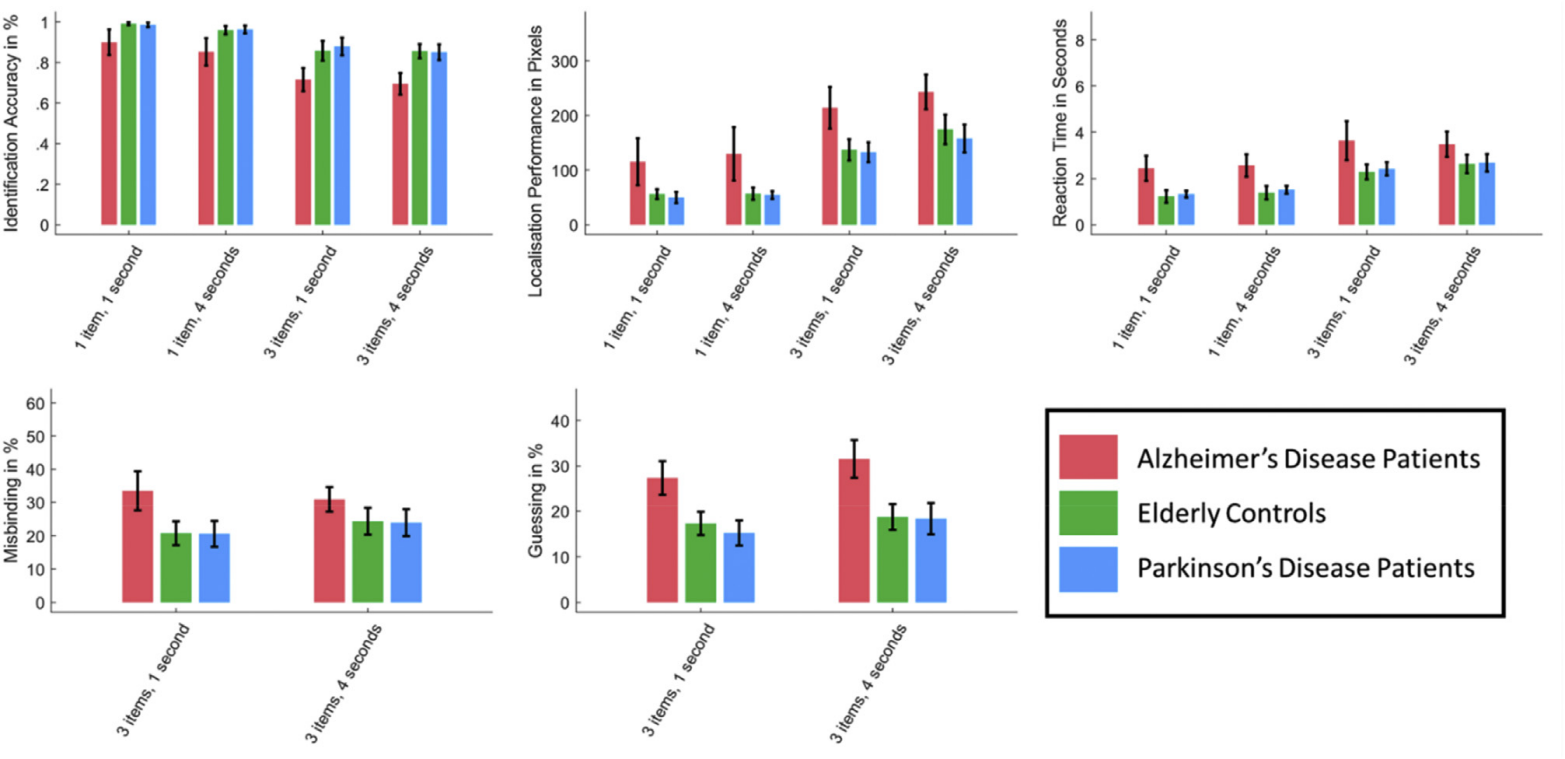
Short-term-memory task results in Alzheimer's and Parkinson's disease compared to controls. Healthy Controls identified the target faster and more often correctly than Alzheimer's Disease Patients, placed the target closer to its original location and guessed and misbound less. Parkinson's patients performed at the same level as controls.

An increase of set size from one to three generally decreased the amount of trials in which targets were identified correctly (F(1,54) = 200.52, *p* < .001, η_p_^2^ = .788; see Fig. 6), increased localisation error F(1,54) = 229.07, *p* < .001, η_p_^2^ = .809) and increased the time taken to identify the target F(1,54) = 107.00, *p* < .001, η_p_^2^ = .665). The increase of delays from one to 4 sec decreased identification accuracy (F(1,54) = 15.81, *p* < .001, η_p_^2^ = .226), increased the error in localising the correct target position (F(1,54) = 31.73, *p* < .001, η_p_^2^ = .370) and increased Guessing (F(1,54) = 31.21, *p* < .001, η_p_^2^ = .366). There were interactions of group and set size in Identification Accuracy (Supplementary Table 11) and of set size and delay in Localisation Performance (Supplementary Table 14) which were followed up on in the Supplementary Materials.

##### 2.7.2.2 Visual imagery in relation to STM performance, brain volumes and cognitive performance

**Fig. 7:**
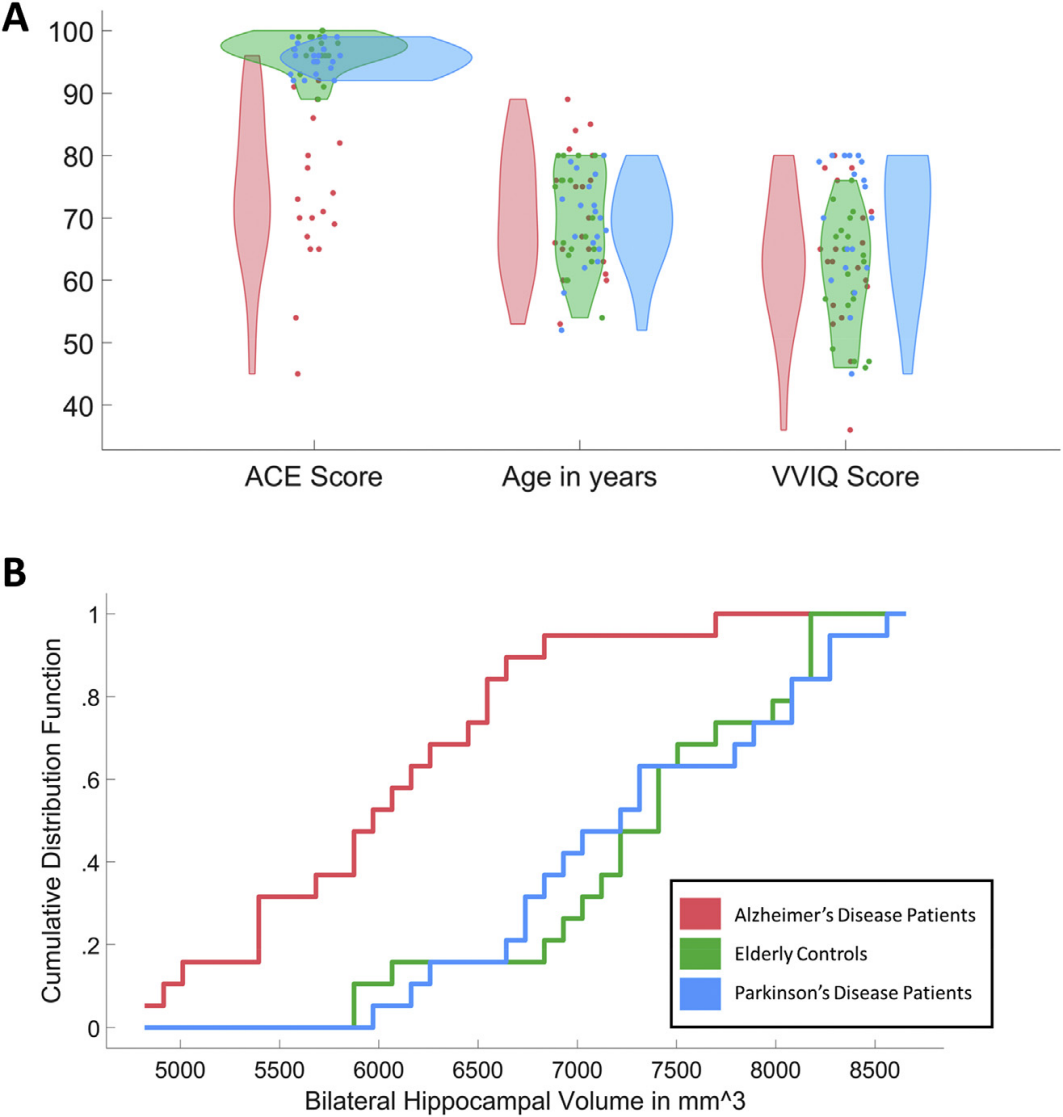
ACE, Age and VVIQ scores and hippocampal volumes in Alzheimer's and Parkinson's disease. In comparison to approximately age-matched controls, there was no significant decrease of VVIQ Scores in Alzheimer's Disease patients (A) despite a decrease of ACE and hippocampal volumes (B). Hippocampal volumes are depicted as cumulative distribution function showing that distributions for Parkinson's disease and Controls are very similar but there is a general shift to smaller volumes in Alzheimer's disease.

As expected, the cognitive screen ACE score (t = 7.14, *p* < .001, Supplementary Table 23), bilateral hippocampal volume (t = 5.52, *p* < .001) and bilateral amygdala volume (t = 2.87, *p* = .007) were significantly decreased in Alzheimer's disease compared to Elderly Controls. Hippocampal volumes were decreased from a mean of 7318.74 mm^3^ (SD = 719.68 mm^3^) in controls to 6017.07 mm^3^ (SD = 733.82) in Alzheimer's disease (Fig. 7, Table 2). There were, however, no such significant differences in the primary visual cortex and the primary motor cortex volumes. But Alzheimer's disease patients showed significantly smaller bilateral fusiform gyrus volumes (17294.94 mm^3^, SD = 1807.01 mm^3^) than elderly controls (18959.33 mm^3^, SD = 1931.04 mm^3^). Parkinson's disease patients were not significantly different from controls on ACE performance or any of these brain volumes (Supplementary Table 24).

On average, healthy controls presented with VVIQ scores of 61.16 (SD = 9.21) of 80 possible points which is not significantly different to Alzheimer's disease patients who scored 63.26 (SD = 11.25); Fig. 7A). So despite their obvious impairment in STM performance and significant reduction in their hippocampal volumes, Alzheimer's disease patients did *not* present with a reduced vividness of visual imagery as indexed by the VVIQ in comparison to healthy controls. This provides further evidence for a potential independence of visual imagery and visual STM. Parkinson's disease patients actually showed slightly significantly increased VVIQ scores (M = 69.31, SD = 10.45) compared to their healthy counterparts (Fig. 7A, t = 2.55, *p* = .015) without any significant hippocampal volume differences in comparison to the latter.

Following up on the significant subfield correlations of CA1, CA3, CA4 and the GC-ML-DG with VVIQ scores in Study 1, we directly compared the volumes of these subfields between the three groups in independent sample *t*-tests. Despite comparable levels of VVIQ scores, all subfields of interest were significantly decreased in Alzheimer's disease patients compared to controls (CA1: t = 4.91, *p* < .001; CA3: t = 6.86, *p* < .001; CA4: t = 6.22, *p* < .001; GC-ML-DG: t = 5.54, *p* < .001). In Parkinson's disease patients, however, there was no such significant difference in comparison to healthy controls (Supplementary Table 25).

These results show that: 1) Profound STM impairment as found in Alzheimer's disease patients is not associated with a significant decrease in self-reported visual imagery as indexed by the VVIQ, and 2) A drastic reduction of hippocampal volume as also found in Alzheimer's disease does not lead to a decrease in self-reported visual imagery.

## 3 Discussion

In our first study, the relationship between visual imagery and visual STM was investigated in healthy participants of a wide age range. In addition, we examined whether there was a correlation between visual imagery and volumes of either the hippocampus and primary visual cortex volumes, two brain regions that have been implicated in studies of visual imagery in prior reports. In the sample studied here, there was no significant correlation between memory performance as measured with a delayed reproduction STM task (Liang et al., 2016; Pertzov et al., 2013; Zokaei, Sillence, et al., 2020) and self-reported visual imagery ability as measured by the VVIQ, suggesting that people's ability to remember object locations and appearances over short periods of time is not predictive of visual imagery. However, visual imagery was significantly correlated to the volumes of both the hippocampus and the primary visual but not the fusiform cortex in healthy people (Fig. 4). Yet, the correlation with the primary visual cortex was relatively small and in the light of the ambiguous literature needs to be taken with caution, especially because the current study focuses on volume and not function.

In a second study, Alzheimer's patients who were expected to show a reduced visual STM performance on the basis of previous studies using delayed reproduction STM tasks (Liang et al., 2016; Zokaei, Sillence, et al., 2020), as well as reduced hippocampal volumes, were assessed to investigate the relationship between visual imagery, STM and hippocampal volume. Alzheimer's disease patients performed significantly worse in the STM task and showed a reduction of hippocampal volume and subfields CA1, CA3, CA4 and GC-ML-DG, but crucially their self-reported visual imagery was intact. Thus, our data shows that, in line with El Haj, Badcock, et al. (2019), Alzheimer's disease patients are unaffected in their vividness of visual imagery. As opposed to previous findings which showed that Parkinson's disease patients score significantly lower on the VVIQ compared to elderly controls (Lo Monaco et al., 2018), our results showed that VVIQ scores were significantly higher in Parkinson's disease patients for the sample tested here.

The imaging results in healthy participants are in line with previously published results linking the vividness of visual imagery to the hippocampus and to the primary visual cortex (Bird et al., 2010; Hassabis et al., 2007). The literature is, however, less conclusive on the subject of a possible link between visual STM and visual imagery. Keogh and Pearson found that performance on their two-forced choice STM task correlated with visual imagery ability in 35 young controls (Keogh & Pearson, 2011). The task was, however, limited to a total of 40 trials. Furthermore, poor imagers were found to perform above chance. A recent study reported significantly worse accuracy on a forced dual choice task in one aphantasic individual, but this was apparent in only the most demanding conditions (Jacobs et al., 2018). On the other hand, Reisberg and colleagues found individuals with high vividness of visual imagery (among a total of 54 participants) performed worse on a STM task, suggesting that their vivid imagery might actually make them more likely to give false-alarm responses (Reisberg, Culver, Heuer, & Fischman, 1986). Moreover, the same group suggested that individuals with vivid imagery, identified in a sample of 14, tend to choose responses more distinct from the target when asked to choose a previously presented colour from a colour array (Heuer, Fischman, & Reisberg, 1986).

In the light of these ambiguous results in the literature, it is worth noting that to the best of our knowledge previous studies that investigated these issues did not examine large samples of participants. Nor did they index recall performance using a continuous STM measure (Bays & Husain, 2008; Ma et al., 2014), as we did here. This type of task has been used to show a significant association between STM metrics and hippocampal atrophy (Liang et al., 2016; Pertzov et al., 2013; Zokaei et al., 2019). Though the first part of the task presented here (identifying the correct of two non-verbalizable fractals) could be solved by capable participants using semantic strategies, the second part of the task will still require them to remember and retrieve spatial information and to somehow translate their mental representation into a visual response.

Although hippocampal volume was related to the vividness of visual imagery in healthy people in the present study, our results do not suggest any association between continuous report measures of STM and the vividness of visual imagery in either healthy controls, Alzheimer's disease or Parkinson's disease. Furthermore, despite significant hippocampal volume loss in Alzheimer's disease there was no significant deterioration in visual imagery in this group. An important caveat though is that visual imagery is, by definition, a self-report measure (VVIQ); we have no reliable objective measure of the vividness of an individual's visual imagery. It is possible that cognitively impaired individuals such as those with Alzheimer's disease might not be reliable at recounting how vivid their visual imagery is currently, and instead might be relying on their abilities in the long distant past, prior to their diagnosis. Another potential issue is the relatively small sample size (*N =* 19) of Alzheimer's disease cases here.

In terms of neuroimaging research on the link between visual imagery and perception, Harrison and Tong (2009) were able to decode orientations of presented Gabor patches in a STM task from early visual cortex over short delays, even in the absence of visual stimuli. These findings support the idea of an involvement of visual imagery in situations in which participants need to hold onto visual information over short periods of time. Albers et al. (2013) were able to take this idea further using an explicit visual imagery task. Their participants were either asked to remember the orientation of a Gabor patch or to visualize a slightly rotated version in their mind's eye. The authors find activity patterns of visual imagery and STM to be identical to those of regular visual perception which would further support the idea of a link between visual imagery and STM and the use of the same or very similar cognitive and anatomical resources for both of them.

Despite these findings, there is some evidence that although identical areas might be involved in imagery and STM, there are also differences in neuroimaging results between these two. Although similar brain areas may be activated in both long-term memory and visual imagery, greater activity may be observed associated with memory than during imagery in parietal control regions and occipital-temporal sensory regions (Slotnick, Thompson, & Kosslyn, 2012). Moreover, the number of different regions was greater than the number of common regions. Based on these findings and their behavioural results, these observations point to important difference between STM and visual imagery, in line with the lack of correlation between STM and imagery in our findings.

Importantly, the work presented here focused on the vividness of visual imagery as measured by the VVIQ. An established questionnaire in the field that can be economically used in large samples. However, the VVIQ is a subjective measure that requires participants to make a critical judgement of their ability to vividly perceive visual stimuli in the absence of appropriate perceptual input. Recent studies have introduced a more objective measure, namely imagery strength: Keogh and Pearson (2011) used a binocular rivalry task to investigate visual imagery in a more detailed fashion. Relying on the fact that one monocular image would become dominant if both eyes were presented with two different images each, the task reveals that previously imagined patterns showed higher probabilities of becoming the dominant pattern in the binocular rivalry presentation (Pearson, Clifford, & Tong, 2008). Future studies should include both, a detailed measure for short-term memory, such as the one presented here, as well as an objective measure for imagery strength, such as the binocular rivalry task. Another future approach would be to relate imagery and short-term memory to the same stimuli in a trial-wise approach as presented by Bona and colleagues (Bona, Cattaneo, Vecchi, Soto, & Silvanto, 2013). Here, participants were to rate the vividness of the memory content as well as the perceived visibility of interference. Such subjective measures could, in the future, be implemented in continuous STM measures.

In summary, the results presented here demonstrate in a large sample of healthy individuals that while there is no association between visual STM and visual imagery, there is a significant correlation between hippocampal and primary visual cortex volumes and visual imagery. Investigation of two different patient groups, Alzheimer's disease and Parkinson's disease, did not reveal any significant relationship either between visual STM and visual imagery, but also failed to show significant loss of visual imagery even with profound hippocampal atrophy in Alzheimer's disease. This might be related to cognitive impairment in Alzheimer's disease affecting self-reported vividness of visual imagery or the relatively small size of cases examined here.

## Author contribution

**Younes Adam Tabi:** Conceptualization; Data curation; Formal analysis; Investigation; Project administration; Methodology; Software; Visualization; Writing - original draft.

**Maria Raquel Maio:** Conceptualization; Data curation; Formal analysis; Investigation; Project administration.

**Bahaaeddin Attaallah:** Data curation.

**Shannon Dickson:** Data curation.

**Daniel Drew:** Data curation.

**Shannon Dickson:** Data curation.

**Mohamad Imran Idris:** Data curation.

**Annika Kienast:** Data curation.

**Verena Klar:** Data curation.

**Lisa Nobis:** Data curation.

**Olivia Plant:** Data curation.

**Youssuf Saleh:** Data curation.

**Timothy Ravinder Sandhu:** Data curation.

**Ellie Slavkova:** Data curation.

**Sofia Toniolo:** Data curation.

**Nahid Zokaei:** Data curation.

**Sanjay G. Manohar:** Methodology; Supervision; Validation; review & editing.

**Masud Husain:** Funding acquisition; Methodology; Resources; Supervision; Validation; review & editing.

## Open practices

The study in this article earned an Open Data badge for transparent practices. Data for this study can be found at: https://osf.io/q37vn/.
