# Supplementary material for: Vividness of visual imagery questionnaire scores and their relationship to visual short-term memory performance
Source: Cortex. 2022 Jan;146:186–99. doi: 10.1016/j.cortex.2021.10.011 (PMC8776564; doi:10.1016/j.cortex.2021.10.011)
Supplement: Multimedia component 1 [file mmc1.docx]

**Supplementary Material**

| **Supplementary Table 1: 2x2-ANOVA of Identification Accuracy in Healthy Controls** | | | | | | | | | | | | | |
| --- | --- | --- | --- | --- | --- | --- | --- | --- | --- | --- | --- | --- | --- |
|  | | **Sum of Squares** | | **df** | | **Mean Square** | | **F** | | **p** | | **η² _p_** | |
| Set size |  | 2.480 |  | 1 |  | 2.480 |  | 721.002 |  | < .001 |  | 0.760 |  |
| Residual |  | 0.784 |  | 228 |  | 0.003 |  |  |  |  |  |  |  |
| Delay |  | 0.114 |  | 1 |  | 0.114 |  | 49.652 |  | < .001 |  | 0.179 |  |
| Residual |  | 0.522 |  | 228 |  | 0.002 |  |  |  |  |  |  |  |
| Set size ✻ Delay |  | 0.021 |  | 1 |  | 0.021 |  | 8.746 |  | 0.003 |  | 0.037 |  |
| Residual |  | 0.543 |  | 228 |  | 0.002 |  |  |  |  |  |  |  |
|  | | | | | | | | | | | | | |
| *Note.*  Type III Sum of Squares | | | | | | | | | | | | | |

| **Supplementary Table 2: Post Hoc Comparisons of Identification Accuracy in Healthy Controls - Set size ✻ Delay** | | | | | | | | | | | |
| --- | --- | --- | --- | --- | --- | --- | --- | --- | --- | --- | --- |
|  | |  | | **Mean Difference** | | **SE** | | **t** | | **p _holm_** | |
| 1 item,four seconds |  | 1 item,one second |  | -0.013 |  | 0.005 |  | -2.821 |  | 0.005 |  |
|  |  | 3 items,four seconds |  | 0.114 |  | 0.005 |  | 22.535 |  | < .001 |  |
|  |  | 3 items,one second |  | 0.082 |  | 0.005 |  | 16.357 |  | < .001 |  |
| 1 item,one second |  | 3 items,four seconds |  | 0.126 |  | 0.005 |  | 25.263 |  | < .001 |  |
|  |  | 3 items,one second |  | 0.095 |  | 0.005 |  | 18.753 |  | < .001 |  |
| 3 items,four seconds |  | 3 items,one second |  | -0.032 |  | 0.005 |  | -7.045 |  | < .001 |  |
|  | | | | | | | | | | | |
| *Note.*  Bonferroni adjusted confidence intervals. | | | | | | | | | | | |

| **Supplementary Table 3: 2x2-ANOVA of Localisation Performance in Healthy Controls** | | | | | | | | | | | | | |
| --- | --- | --- | --- | --- | --- | --- | --- | --- | --- | --- | --- | --- | --- |
|  | | **Sum of Squares** | | **df** | | **Mean Square** | | **F** | | **p** | | **η² _p_** | |
| Set size |  | 2.177e +6 |  | 1 |  | 2.177e +6 |  | 1154.662 |  | < .001 |  | 0.835 |  |
| Residual |  | 429796.004 |  | 228 |  | 1885.070 |  |  |  |  |  |  |  |
| Delay |  | 38691.151 |  | 1 |  | 38691.151 |  | 54.287 |  | < .001 |  | 0.192 |  |
| Residual |  | 162498.491 |  | 228 |  | 712.713 |  |  |  |  |  |  |  |
| Set size ✻ Delay |  | 22362.033 |  | 1 |  | 22362.033 |  | 31.986 |  | < .001 |  | 0.123 |  |
| Residual |  | 159400.731 |  | 228 |  | 699.126 |  |  |  |  |  |  |  |
|  | | | | | | | | | | | | | |
| *Note.*  Type III Sum of Squares | | | | | | | | | | | | | |

| **Supplementary Table 4: Post Hoc Comparisons of Localisation Performance in Healthy Controls - Set size ✻ Delay** | | | | | | | | | | | | | | | |  |
| --- | --- | --- | --- | --- | --- | --- | --- | --- | --- | --- | --- | --- | --- | --- | --- | --- |
|  | | |  | | | | **Mean Difference** | | | | **SE** | | **t** | | **p _holm_** | |
| one item,four seconds | |  | one item,one second | | |  | 3.117 | | |  | 2.483 |  | 1.255 |  | 0.210 |  |
|  | |  | three items,four seconds | | |  | -107.375 | | |  | 3.359 |  | -31.964 |  | < .001 |  |
|  | |  | three items,one second | | |  | -84.495 | | |  | 3.368 |  | -25.087 |  | < .001 |  |
| one item,one second | |  | three items,four seconds | | |  | -110.491 | | |  | 3.368 |  | -32.805 |  | < .001 |  |
|  | |  | three items,one second | | |  | -87.611 | | |  | 3.359 |  | -26.080 |  | < .001 |  |
| three items,four seconds | |  | three items,one second | | |  | 22.880 | | |  | 2.483 |  | 9.215 |  | < .001 |  |
|  | | | | | | | | | | | | | | | | |
| *Note.*  Bonferroni adjusted confidence intervals. | | | | | | | | | | | | | | | | |
| **Supplementary Table 5: 2-ANOVA of Misbinding in Healthy Controls** | | | | | | | | | | | | | | | |  |
|  | | **Sum of Squares** | | **df** | | **Mean Square** | | **F** | | **p** | | | | **η² _p_** | |  |
| Delay |  | 0.060 |  | 1 |  | 0.060 |  | 10.951 |  | 0.001 | | |  | 0.048 |  |  |
| Residual |  | 1.181 |  | 217 |  | 0.005 |  |  |  |  | | |  |  |  |  |
|  | | | | | | | | | | | | | | | |  |
| *Note.*  Type III Sum of Squares | | | | | | | | | | | | | | | |  |

| **Supplementary Table 6: 2-ANOVA of Guessing in Healthy Controls** | | | | | | | | | | | | | |
| --- | --- | --- | --- | --- | --- | --- | --- | --- | --- | --- | --- | --- | --- |
|  | | **Sum of Squares** | | **df** | | **Mean Square** | | **F** | | **p** | | **η² _p_** | |
| Delay |  | 0.012 |  | 1 |  | 0.012 |  | 20.414 |  | < .001 |  | 0.082 |  |
| Residual |  | 0.129 |  | 228 |  | 5.654e -4 |  |  |  |  |  |  |  |
|  | | | | | | | | | | | | | |
| *Note.*  Type III Sum of Squares | | | | | | | | | | | | | |

| **Supplementary Table 7: 2x2-ANOVA of Reaction Time in Healthy Controls** | | | | | | | | | | | | | |
| --- | --- | --- | --- | --- | --- | --- | --- | --- | --- | --- | --- | --- | --- |
|  | | **Sum of Squares** | | **df** | | **Mean Square** | | **F** | | **p** | | **η² _p_** | |
| Set size |  | 333.604 |  | 1 |  | 333.604 |  | 1183.213 |  | < .001 |  | 0.838 |  |
| Residual |  | 64.284 |  | 228 |  | 0.282 |  |  |  |  |  |  |  |
| Delay |  | 22.579 |  | 1 |  | 22.579 |  | 210.420 |  | < .001 |  | 0.480 |  |
| Residual |  | 24.466 |  | 228 |  | 0.107 |  |  |  |  |  |  |  |
| Set size ✻ Delay |  | 4.295 |  | 1 |  | 4.295 |  | 40.786 |  | < .001 |  | 0.152 |  |
| Residual |  | 24.011 |  | 228 |  | 0.105 |  |  |  |  |  |  |  |
|  | | | | | | | | | | | | | |
| *Note.*  Type III Sum of Squares | | | | | | | | | | | | | |

| **Supplementary Table 8: Post Hoc Comparisons of Reaction Time in Healthy Controls - Set size ✻ Delay** | | | | | | | | | | | |
| --- | --- | --- | --- | --- | --- | --- | --- | --- | --- | --- | --- |
|  | |  | | **Mean Difference** | | **SE** | | **t** | | **p _holm_** | |
| one item,four seconds |  | one item,one second |  | 0.177 |  | 0.030 |  | 5.811 |  | < .001 |  |
|  |  | three items,four seconds |  | -1.344 |  | 0.041 |  | -32.681 |  | < .001 |  |
|  |  | three items,one second |  | -0.893 |  | 0.041 |  | -21.659 |  | < .001 |  |
| one item,one second |  | three items,four seconds |  | -1.521 |  | 0.041 |  | -36.891 |  | < .001 |  |
|  |  | three items,one second |  | -1.070 |  | 0.041 |  | -26.020 |  | < .001 |  |
| three items,four seconds |  | three items,one second |  | 0.451 |  | 0.030 |  | 14.800 |  | < .001 |  |
|  | | | | | | | | | | | |
| *Note.*  Bonferroni adjusted confidence intervals. | | | | | | | | | | | |

| **Supplementary Table 9: Correlations of VVIQ with variables of interest in Healthy Controls** | | | | | |
| --- | --- | --- | --- | --- | --- |
|  | |  | | **VVIQ** | |
| VVIQ_Total_T1 |  | Pearson's r |  | — |  |
|  |  | p-value |  | — |  |
| Age |  | Pearson's r |  | 0.088 |  |
|  |  | p-value |  | 0.186 |  |
| ACE |  | Pearson's r |  | 0.069 |  |
|  |  | p-value |  | 0.560 |  |
| Identification Accuracy |  | Pearson's r |  | 0.007 |  |
|  |  | p-value |  | 0.916 |  |
| Localisation Performance |  | Pearson's r |  | -0.020 |  |
|  |  | p-value |  | 0.769 |  |
| Misbinding |  | Pearson's r |  | -0.041 |  |
|  |  | p-value |  | 0.535 |  |
| Guessing |  | Pearson's r |  | -0.107 |  |
|  |  | p-value |  | 0.107 |  |
| MeanRT |  | Pearson's r |  | -0.021 |  |
|  |  | p-value |  | 0.757 |  |
| Bilateral Hippocampal Volume |  | Pearson's r |  | 0.348 | ** |
|  |  | p-value |  | 0.009 |  |
| Left Hippocampal Volume |  | Pearson's r |  | 0.329 | * |
|  |  | p-value |  | 0.013 |  |
| Right Hippocampal Volume |  | Pearson's r |  | 0.263 | * |
|  |  | p-value |  | 0.050 |  |
| Bilateral Amygdala Volume |  | Pearson's r |  | 0.089 |  |
|  |  | p-value |  | 0.513 |  |
| Left Amygdala Volume |  | Pearson's r |  | 0.045 |  |
|  |  | p-value |  | 0.745 |  |
| Right Amygdala Volume |  | Pearson's r |  | 0.108 |  |
|  |  | p-value |  | 0.427 |  |
| Bilateral Motor Cortex Volume |  | Pearson's r |  | -0.052 |  |
|  |  | p-value |  | 0.704 |  |
| Left Motor Cortex Volume |  | Pearson's r |  | -0.013 |  |
|  |  | p-value |  | 0.926 |  |
| Right Motor Cortex Volume |  | Pearson's r |  | -0.085 |  |
|  |  | p-value |  | 0.533 |  |
| Bilateral Visual Cortex Volume |  | Pearson's r |  | 0.263 |  |
|  |  | p-value |  | 0.050 |  |
| Left Visual Cortex Volume |  | Pearson's r |  | 0.287 | * |
|  |  | p-value |  | 0.032 |  |
| Right Visual Cortex Volume |  | Pearson's r |  | 0.217 |  |
|  |  | p-value |  | 0.107 |  |
| Bilateral Fusiform Cortex Volume |  | Pearson's r |  | 0.204 |  |
|  |  | p-value |  | 0.132 |  |
| Left Fusiform Cortex Volume |  | Pearson's r |  | 0.194 |  |
|  |  | p-value |  | 0.153 |  |
| Right Fusiform Cortex Volume |  | Pearson's r |  | 0.173 |  |
|  |  | p-value |  | 0.201 |  |

| **Supplementary Table 10: Correlations of VVIQ Score with Hippocampal Subfields** | | | | | |
| --- | --- | --- | --- | --- | --- |
| **Variable** | |  | | **VVIQ Score** | |
| VVIQ Score |  | Pearson's r |  | — |  |
|  |  | p-value |  | — |  |
| Hippocampal Tail |  | Pearson's r |  | 0.194 |  |
|  |  | p-value |  | 0.164 |  |
| Subiculum |  | Pearson's r |  | 0.249 |  |
|  |  | p-value |  | 0.072 |  |
| CA1 |  | Pearson's r |  | 0.421 | ** |
|  |  | p-value |  | 0.002 |  |
| Hippocampal Fissure |  | Pearson's r |  | 0.093 |  |
|  |  | p-value |  | 0.508 |  |
| Presubiculum |  | Pearson's r |  | 0.177 |  |
|  |  | p-value |  | 0.204 |  |
| Parasubiculum |  | Pearson's r |  | 0.211 |  |
|  |  | p-value |  | 0.129 |  |
| Molecular Layer |  | Pearson's r |  | 0.129 |  |
|  |  | p-value |  | 0.358 |  |
| GC-ML-DG |  | Pearson's r |  | 0.470 | *** |
|  |  | p-value |  | < .001 |  |
| CA3 |  | Pearson's r |  | 0.416 | ** |
|  |  | p-value |  | 0.002 |  |
| CA4 |  | Pearson's r |  | 0.468 | *** |
|  |  | p-value |  | < .001 |  |
| Fimbria |  | Pearson's r |  | 0.261 |  |
|  |  | p-value |  | 0.059 |  |
| HATA |  | Pearson's r |  | 0.041 |  |
|  |  | p-value |  | 0.773 |  |

| **Supplementary Table 11: 2x2x2-ANOVA of Set size, Delay and Group Effects on Identification Accuracy in Elderly Controls vs. Parkinson’s Disease Patients vs. Alzheimer’s Disease Patients** | | | | | | | | | | | | | |
| --- | --- | --- | --- | --- | --- | --- | --- | --- | --- | --- | --- | --- | --- |
| **Cases** | | **Sum of Squares** | | **df** | | **Mean Square** | | **F** | | **p** | | **η² _p_** | |
| Set size |  | 1.009 |  | 1 |  | 1.009 |  | 200.519 |  | < .001 |  | 0.788 |  |
| Set size ✻ groupnum |  | 0.042 |  | 2 |  | 0.021 |  | 4.165 |  | 0.021 |  | 0.134 |  |
| Residuals |  | 0.272 |  | 54 |  | 0.005 |  |  |  |  |  |  |  |
| Delay |  | 0.037 |  | 1 |  | 0.037 |  | 15.805 |  | < .001 |  | 0.226 |  |
| Delay ✻ groupnum |  | 0.003 |  | 2 |  | 0.001 |  | 0.626 |  | 0.538 |  | 0.023 |  |
| Residuals |  | 0.126 |  | 54 |  | 0.002 |  |  |  |  |  |  |  |
| Set size ✻ Delay |  | 0.004 |  | 1 |  | 0.004 |  | 1.227 |  | 0.273 |  | 0.022 |  |
| Set size ✻ Delay ✻ groupnum |  | 0.004 |  | 2 |  | 0.002 |  | 0.531 |  | 0.591 |  | 0.019 |  |
| Residuals |  | 0.180 |  | 54 |  | 0.003 |  |  |  |  |  |  |  |
|  | | | | | | | | | | | | | |
| Note.  Type III Sum of Squares | | | | | | | | | | | | | |

| **Between Subjects Effects** | | | | | | | | | | | | | |
| --- | --- | --- | --- | --- | --- | --- | --- | --- | --- | --- | --- | --- | --- |
| **Cases** | | **Sum of Squares** | | **df** | | **Mean Square** | | **F** | | **p** | | **η² _p_** | |
| groupnum |  | 0.820 |  | 2 |  | 0.410 |  | 18.912 |  | < .001 |  | 0.412 |  |
| Residuals |  | 1.171 |  | 54 |  | 0.022 |  |  |  |  |  |  |  |
|  | | | | | | | | | | | | | |
| Note.  Type III Sum of Squares | | | | | | | | | | | | | |

| **Supplementary Table 12: Post-hoc t-tests following up on the interaction between group and set size of Identification Accuracy in Elderly Controls vs. Parkinson’s Disease Patients vs. Alzheimer’s Disease Patients** | | | | | | | | | | | |
| --- | --- | --- | --- | --- | --- | --- | --- | --- | --- | --- | --- |
|  | |  | | **Mean Difference** | | **SE** | | **t** | | **p _holm_** | |
| Elderly, Controls, one.item |  | Alzheimer's, Disease, Patients, one.item |  | 0.099 |  | 0.027 |  | 3.738 |  | 0.002 |  |
|  |  | Parkinson's, Disease, Patients, one.item |  | 8.772e -4 |  | 0.027 |  | 0.033 |  | 1.000 |  |
|  |  | Elderly, Controls, three.items |  | 0.118 |  | 0.016 |  | 7.277 |  | < .001 |  |
|  |  | Alzheimer's, Disease, Patients, three.items |  | 0.270 |  | 0.027 |  | 10.190 |  | < .001 |  |
|  |  | Parkinson's, Disease, Patients, three.items |  | 0.111 |  | 0.027 |  | 4.169 |  | < .001 |  |
| Alzheimer's, Disease, Patients, one.item |  | Parkinson's, Disease, Patients, one.item |  | -0.098 |  | 0.027 |  | -3.705 |  | 0.002 |  |
|  |  | Elderly, Controls, three.items |  | 0.019 |  | 0.027 |  | 0.728 |  | 1.000 |  |
|  |  | Alzheimer's, Disease, Patients, three.items |  | 0.171 |  | 0.016 |  | 10.511 |  | < .001 |  |
|  |  | Parkinson's, Disease, Patients, three.items |  | 0.011 |  | 0.027 |  | 0.430 |  | 1.000 |  |
| Parkinson's, Disease, Patients, one.item |  | Elderly, Controls, three.items |  | 0.118 |  | 0.027 |  | 4.433 |  | < .001 |  |
|  |  | Alzheimer's, Disease, Patients, three.items |  | 0.269 |  | 0.027 |  | 10.157 |  | < .001 |  |
|  |  | Parkinson's, Disease, Patients, three.items |  | 0.110 |  | 0.016 |  | 6.738 |  | < .001 |  |
| Elderly, Controls, three.items |  | Alzheimer's, Disease, Patients, three.items |  | 0.152 |  | 0.027 |  | 5.723 |  | < .001 |  |
|  |  | Parkinson's, Disease, Patients, three.items |  | -0.008 |  | 0.027 |  | -0.298 |  | 1.000 |  |
| Alzheimer's, Disease, Patients, three.items |  | Parkinson's, Disease, Patients, three.items |  | -0.160 |  | 0.027 |  | -6.021 |  | < .001 |  |
|  | | | | | | | | | | | |
| Note.  P-value adjusted for comparing a family of 15 | | | | | | | | | | | |
| Note.  Results are averaged over the levels of: Delay | | | | | | | | | | | |

| **Supplementary Table 13: Post-hoc t-tests following up on the main effect of group of Identification Accuracy in Elderly Controls vs. Parkinson’s Disease Patients vs. Alzheimer’s Disease Patients** | | | | | | | | | | | |
| --- | --- | --- | --- | --- | --- | --- | --- | --- | --- | --- | --- |
|  | |  | | **Mean Difference** | | **SE** | | **t** | | **p _holm_** | |
| Elderly, Controls |  | Alzheimer's, Disease, Patients |  | 0.125 |  | 0.024 |  | 5.251 |  | < .001 |  |
|  |  | Parkinson's, Disease, Patients |  | -0.004 |  | 0.024 |  | -0.147 |  | 0.884 |  |
| Alzheimer's, Disease, Patients |  | Parkinson's, Disease, Patients |  | -0.129 |  | 0.024 |  | -5.398 |  | < .001 |  |
|  | | | | | | | | | | | |
| Note.  P-value adjusted for comparing a family of 3 | | | | | | | | | | | |
| Note.  Results are averaged over the levels of: Set size, Delay | | | | | | | | | | | |

| **Supplementary Table 14: 2x2x2-ANOVA of Set size, Delay and Group Effects on Localisation Performance in Elderly Controls vs. Parkinson’s Disease Patients vs. Alzheimer’s Disease Patients** | | | | | | | | | | | | | |
| --- | --- | --- | --- | --- | --- | --- | --- | --- | --- | --- | --- | --- | --- |
| **Cases** | | **Sum of Squares** | | **df** | | **Mean Square** | | **F** | | **p** | | **η² _p_** | |
| Set size |  | 564008.525 |  | 1 |  | 564008.525 |  | 229.066 |  | < .001 |  | 0.809 |  |
| Set size ✻ groupnum |  | 1624.876 |  | 2 |  | 812.438 |  | 0.330 |  | 0.720 |  | 0.012 |  |
| Residuals |  | 132959.280 |  | 54 |  | 2462.209 |  |  |  |  |  |  |  |
| Delay |  | 19573.247 |  | 1 |  | 19573.247 |  | 31.733 |  | < .001 |  | 0.370 |  |
| Delay ✻ groupnum |  | 456.374 |  | 2 |  | 228.187 |  | 0.370 |  | 0.693 |  | 0.014 |  |
| Residuals |  | 33307.534 |  | 54 |  | 616.806 |  |  |  |  |  |  |  |
| Set size ✻ Delay |  | 8259.707 |  | 1 |  | 8259.707 |  | 10.010 |  | 0.003 |  | 0.156 |  |
| Set size ✻ Delay ✻ groupnum |  | 1221.526 |  | 2 |  | 610.763 |  | 0.740 |  | 0.482 |  | 0.027 |  |
| Residuals |  | 44556.363 |  | 54 |  | 825.118 |  |  |  |  |  |  |  |
|  | | | | | | | | | | | | | |
| Note.  Type III Sum of Squares | | | | | | | | | | | | | |

| **Between Subjects Effects** | | | | | | | | | | | | | |
| --- | --- | --- | --- | --- | --- | --- | --- | --- | --- | --- | --- | --- | --- |
| **Cases** | | **Sum of Squares** | | **df** | | **Mean Square** | | **F** | | **p** | | **η² _p_** | |
| groupnum |  | 273025.422 |  | 2 |  | 136512.711 |  | 14.798 |  | < .001 |  | 0.354 |  |
| Residuals |  | 498141.830 |  | 54 |  | 9224.849 |  |  |  |  |  |  |  |
|  | | | | | | | | | | | | | |
| Note.  Type III Sum of Squares | | | | | | | | | | | | | |

| **Supplementary Table 15: Post-hoc t-tests following up on the interaction between group and set size of Localisation Performance in Elderly Controls vs. Parkinson’s Disease Patients vs. Alzheimer’s Disease Patients Delay*Set size** | | | | | | | | | | | |
| --- | --- | --- | --- | --- | --- | --- | --- | --- | --- | --- | --- |
|  | |  | | **Mean Difference** | | **SE** | | **t** | | **p _holm_** | |
| three.items, one.second |  | one.item, one.second |  | 87.435 |  | 7.594 |  | 11.513 |  | < .001 |  |
|  |  | three.items, four.seconds |  | -30.569 |  | 5.030 |  | -6.078 |  | < .001 |  |
|  |  | one.item, four.seconds |  | 80.942 |  | 7.350 |  | 11.013 |  | < .001 |  |
| one.item, one.second |  | three.items, four.seconds |  | -118.004 |  | 7.350 |  | -16.056 |  | < .001 |  |
|  |  | one.item, four.seconds |  | -6.493 |  | 5.030 |  | -1.291 |  | 0.200 |  |
| three.items, four.seconds |  | one.item, four.seconds |  | 111.511 |  | 7.594 |  | 14.684 |  | < .001 |  |
|  | | | | | | | | | | | |
| Note.  P-value adjusted for comparing a family of 6 | | | | | | | | | | | |
| Note.  Results are averaged over the levels of: groupnum | | | | | | | | | | | |

| **Supplementary Table 16: Post-hoc t-tests following up on the main effect of group of Localisation Performance in Elderly Controls vs. Parkinson’s Disease Patients vs. Alzheimer’s Disease Patients** | | | | | | | | | | | |
| --- | --- | --- | --- | --- | --- | --- | --- | --- | --- | --- | --- |
|  | |  | | **Mean Difference** | | **SE** | | **t** | | **p _holm_** | |
| Elderly, Controls |  | Alzheimer's, Disease, Patients |  | -69.445 |  | 15.581 |  | -4.457 |  | < .001 |  |
|  |  | Parkinson's, Disease, Patients |  | 7.370 |  | 15.581 |  | 0.473 |  | 0.638 |  |
| Alzheimer's, Disease, Patients |  | Parkinson's, Disease, Patients |  | 76.814 |  | 15.581 |  | 4.930 |  | < .001 |  |
|  | | | | | | | | | | | |
| Note.  P-value adjusted for comparing a family of 3 | | | | | | | | | | | |
| Note.  Results are averaged over the levels of: Set size, Delay | | | | | | | | | | | |

| **Supplementary Table 17: 2x2-ANOVA of Delay and Group Effects on Misbinding in Elderly Controls vs. Parkinson’s Disease Patients vs. Alzheimer’s Disease Patients** | | | | | | | | | | | | | |
| --- | --- | --- | --- | --- | --- | --- | --- | --- | --- | --- | --- | --- | --- |
| **Cases** | | **Sum of Squares** | | **df** | | **Mean Square** | | **F** | | **p** | | **η² _p_** | |
| Delay |  | 0.006 |  | 1 |  | 0.006 |  | 1.076 |  | 0.304 |  | 0.020 |  |
| Delay ✻ groupnum |  | 0.023 |  | 2 |  | 0.012 |  | 2.061 |  | 0.137 |  | 0.071 |  |
| Residuals |  | 0.306 |  | 54 |  | 0.006 |  |  |  |  |  |  |  |
|  | | | | | | | | | | | | | |
| Note.  Type III Sum of Squares | | | | | | | | | | | | | |

| **Between Subjects Effects** | | | | | | | | | | | | | |
| --- | --- | --- | --- | --- | --- | --- | --- | --- | --- | --- | --- | --- | --- |
| **Cases** | | **Sum of Squares** | | **df** | | **Mean Square** | | **F** | | **p** | | **η² _p_** | |
| groupnum |  | 0.245 |  | 2 |  | 0.122 |  | 12.297 |  | < .001 |  | 0.313 |  |
| Residuals |  | 0.537 |  | 54 |  | 0.010 |  |  |  |  |  |  |  |
|  | | | | | | | | | | | | | |
| Note.  Type III Sum of Squares | | | | | | | | | | | | | |

| **Supplementary Table 18: Post-hoc t-tests following up on the main effect of group on Misbinding in Elderly Controls vs. Parkinson’s Disease Patients vs. Alzheimer’s Disease Patients** | | | | | | | | | | | |
| --- | --- | --- | --- | --- | --- | --- | --- | --- | --- | --- | --- |
|  | |  | | **Mean Difference** | | **SE** | | **t** | | **p _holm_** | |
| Elderly, Controls |  | Alzheimer's, Disease, Patients |  | -0.097 |  | 0.023 |  | -4.227 |  | < .001 |  |
|  |  | Parkinson's, Disease, Patients |  | 0.003 |  | 0.023 |  | 0.133 |  | 0.895 |  |
| Alzheimer's, Disease, Patients |  | Parkinson's, Disease, Patients |  | 0.100 |  | 0.023 |  | 4.360 |  | < .001 |  |
|  | | | | | | | | | | | |
| Note.  P-value adjusted for comparing a family of 3 | | | | | | | | | | | |
| Note.  Results are averaged over the levels of: Delay | | | | | | | | | | | |

| **Supplementary Table 19: 2x2-ANOVA of Delay and Group Effects on Guessing in Elderly Controls vs. Parkinson’s Disease Patients vs. Alzheimer’s Disease Patients** | | | | | | | | | | | | | |
| --- | --- | --- | --- | --- | --- | --- | --- | --- | --- | --- | --- | --- | --- |
| **Cases** | | **Sum of Squares** | | **df** | | **Mean Square** | | **F** | | **p** | | **η² _p_** | |
| Delay |  | 0.024 |  | 1 |  | 0.024 |  | 31.207 |  | < .001 |  | 0.366 |  |
| Delay ✻ groupnum |  | 0.004 |  | 2 |  | 0.002 |  | 2.388 |  | 0.101 |  | 0.081 |  |
| Residuals |  | 0.042 |  | 54 |  | 7.815e -4 |  |  |  |  |  |  |  |
|  | | | | | | | | | | | | | |
| Note.  Type III Sum of Squares | | | | | | | | | | | | | |

| **Between Subjects Effects** | | | | | | | | | | | | | |
| --- | --- | --- | --- | --- | --- | --- | --- | --- | --- | --- | --- | --- | --- |
| **Cases** | | **Sum of Squares** | | **df** | | **Mean Square** | | **F** | | **p** | | **η² _p_** | |
| groupnum |  | 0.369 |  | 2 |  | 0.184 |  | 21.583 |  | < .001 |  | 0.444 |  |
| Residuals |  | 0.461 |  | 54 |  | 0.009 |  |  |  |  |  |  |  |
|  | | | | | | | | | | | | | |
| Note.  Type III Sum of Squares | | | | | | | | | | | | | |

| **Supplementary Table 20: Post-hoc t-tests following up on the main effect of group on Guessing in Elderly Controls vs. Parkinson’s Disease Patients vs. Alzheimer’s Disease Patients** | | | | | | | | | | | |
| --- | --- | --- | --- | --- | --- | --- | --- | --- | --- | --- | --- |
|  | |  | | **Mean Difference** | | **SE** | | **t** | | **p _holm_** | |
| Elderly, Controls |  | Alzheimer's, Disease, Patients |  | -0.114 |  | 0.021 |  | -5.375 |  | < .001 |  |
|  |  | Parkinson's, Disease, Patients |  | 0.012 |  | 0.021 |  | 0.585 |  | 0.561 |  |
| Alzheimer's, Disease, Patients |  | Parkinson's, Disease, Patients |  | 0.126 |  | 0.021 |  | 5.960 |  | < .001 |  |
|  | | | | | | | | | | | |
| Note.  P-value adjusted for comparing a family of 3 | | | | | | | | | | | |
| Note.  Results are averaged over the levels of: Delay | | | | | | | | | | | |

| **Supplementary Table 21: 2x2x2-ANOVA of Set size, Delay and Group Effects on Reaction Time in Elderly Controls vs. Parkinson’s Disease Patients vs. Alzheimer’s Disease Patients** | | | | | | | | | | | | | |
| --- | --- | --- | --- | --- | --- | --- | --- | --- | --- | --- | --- | --- | --- |
| **Cases** | | **Sum of Squares** | | **df** | | **Mean Square** | | **F** | | **p** | | **η² _p_** | |
| Set size |  | 70.762 |  | 1 |  | 70.762 |  | 107.001 |  | < .001 |  | 0.665 |  |
| Set size ✻ groupnum |  | 0.088 |  | 2 |  | 0.044 |  | 0.066 |  | 0.936 |  | 0.002 |  |
| Residuals |  | 35.711 |  | 54 |  | 0.661 |  |  |  |  |  |  |  |
| Delay |  | 1.379 |  | 1 |  | 1.379 |  | 3.279 |  | 0.076 |  | 0.057 |  |
| Delay ✻ groupnum |  | 0.839 |  | 2 |  | 0.420 |  | 0.998 |  | 0.375 |  | 0.036 |  |
| Residuals |  | 22.703 |  | 54 |  | 0.420 |  |  |  |  |  |  |  |
| Set size ✻ Delay |  | 0.002 |  | 1 |  | 0.002 |  | 0.011 |  | 0.918 |  | 1.961e -4 |  |
| Set size ✻ Delay ✻ groupnum |  | 0.559 |  | 2 |  | 0.279 |  | 1.637 |  | 0.204 |  | 0.057 |  |
| Residuals |  | 9.213 |  | 54 |  | 0.171 |  |  |  |  |  |  |  |
|  | | | | | | | | | | | | | |
| Note.  Type III Sum of Squares | | | | | | | | | | | | | |

| **Between Subjects Effects** | | | | | | | | | | | | | |
| --- | --- | --- | --- | --- | --- | --- | --- | --- | --- | --- | --- | --- | --- |
| **Cases** | | **Sum of Squares** | | **df** | | **Mean Square** | | **F** | | **p** | | **η² _p_** | |
| groupnum |  | 61.758 |  | 2 |  | 30.879 |  | 15.768 |  | < .001 |  | 0.369 |  |
| Residuals |  | 105.751 |  | 54 |  | 1.958 |  |  |  |  |  |  |  |
|  | | | | | | | | | | | | | |
| Note.  Type III Sum of Squares | | | | | | | | | | | | | |

| **Supplementary Table 22: Post-hoc t-tests following up on the main effect of group on Reaction Time in Elderly Controls vs. Parkinson’s Disease Patients vs. Alzheimer’s Disease Patients** | | | | | | | | | | | |
| --- | --- | --- | --- | --- | --- | --- | --- | --- | --- | --- | --- |
|  | |  | | **Mean Difference** | | **SE** | | **t** | | **p _holm_** | |
| Elderly, Controls |  | Alzheimer's, Disease, Patients |  | -1.152 |  | 0.227 |  | -5.073 |  | < .001 |  |
|  |  | Parkinson's, Disease, Patients |  | -0.102 |  | 0.227 |  | -0.450 |  | 0.655 |  |
| Alzheimer's, Disease, Patients |  | Parkinson's, Disease, Patients |  | 1.049 |  | 0.227 |  | 4.623 |  | < .001 |  |
|  | | | | | | | | | | | |
| Note.  P-value adjusted for comparing a family of 3 | | | | | | | | | | | |
| Note.  Results are averaged over the levels of: Set size, Delay | | | | | | | | | | | |

| **Supplementary Table 23: Independent sample t-tests for the variables of interest in comparison between Elderly Controls and Alzheimer’s Disease Patients** | | | | | | | | | |
| --- | --- | --- | --- | --- | --- | --- | --- | --- | --- |
|  | | **Test** | | **Statistic** | | **df** | | **p** | |
| ACE |  | Student |  | 7.135 |  | 36 |  | < .001 |  |
|  |  | Mann-Whitney |  | 351.000 |  |  |  | < .001 |  |
| Age |  | Student |  | -0.247 |  | 36 |  | 0.806 |  |
|  |  | Mann-Whitney |  | 174.500 |  |  |  | 0.872 |  |
| VVIQ |  | Student |  | -0.631 |  | 36 |  | 0.532 |  |
|  |  | Mann-Whitney |  | 160.000 |  |  |  | 0.559 |  |
| Bilateral Hippocampal Volume |  | Student |  | 5.520 |  | 36 |  | < .001 |  |
|  |  | Mann-Whitney |  | 322.000 |  |  |  | < .001 |  |
| Bilateral Amygdala Volume |  | Student |  | 2.867 |  | 36 |  | 0.007 |  |
|  |  | Mann-Whitney |  | 270.000 |  |  |  | 0.009 |  |
| Bilateral Primary Motor Cortex Volume |  | Student |  | -0.685 |  | 36 |  | 0.498 |  |
|  |  | Mann-Whitney |  | 143.000 |  |  |  | 0.280 |  |
| Bilateral Primary Visual Cortex Volume |  | Student |  | -0.040 |  | 36 |  | 0.969 |  |
|  |  | Mann-Whitney |  | 164.000 |  |  |  | 0.640 |  |
| Bilateral Fusiform Cortex Volume |  | Student |  | 2.743 |  | 36 |  | 0.009 |  |
|  |  | Mann-Whitney |  | 274.000 |  |  |  | 0.007 |  |
|  | | | | | | | | | |

| **Supplementary Table 24: Independent sample t-tests for the variables of interest in comparison between Elderly Controls and Parkinson’s Disease Patients** | | | | | | | | | |
| --- | --- | --- | --- | --- | --- | --- | --- | --- | --- |
|  | | **Test** | | **Statistic** | | **df** | | **p** | |
| ACE |  | Student |  | 1.274 |  | 36 |  | 0.211 |  |
|  |  | Mann-Whitney |  | 236.500 |  |  |  | 0.102 |  |
| Age |  | Student |  | 0.455 |  | 36 |  | 0.652 |  |
|  |  | Mann-Whitney |  | 195.500 |  |  |  | 0.671 |  |
| VVIQ |  | Student |  | -2.553 |  | 36 |  | 0.015 |  |
|  |  | Mann-Whitney |  | 100.000 |  |  |  | 0.019 |  |
| Bilateral Hippocampal Volume |  | Student |  | -0.050 |  | 36 |  | 0.961 |  |
|  |  | Mann-Whitney |  | 189.000 |  |  |  | 0.815 |  |
| Bilateral Amygdala Volume |  | Student |  | -1.533 |  | 36 |  | 0.134 |  |
|  |  | Mann-Whitney |  | 117.000 |  |  |  | 0.066 |  |
| Bilateral Primary Motor Cortex Volume |  | Student |  | 0.050 |  | 36 |  | 0.960 |  |
|  |  | Mann-Whitney |  | 165.000 |  |  |  | 0.661 |  |
| Bilateral Primary Visual Cortex Volume |  | Student |  | 0.341 |  | 36 |  | 0.735 |  |
|  |  | Mann-Whitney |  | 175.000 |  |  |  | 0.884 |  |
| Bilateral Fusiform Cortex Volume |  | Student |  | 0.449 |  | 36 |  | 0.621 |  |
|  |  | Mann-Whitney |  | 203.000 |  |  |  | 0.521 |  |
|  | | | | | | | | | |

| **Table 25: Independent sample t-tests for hippocampal subfields in comparison between Elderly Controls and Parkinson’s Disease Patients** | | | | | | | |
| --- | --- | --- | --- | --- | --- | --- | --- |
|  | | **t** | | **df** | | **p** | |
| bilateral_CA1_ |  | -0.893 |  | 36 |  | 0.378 |  |
| bilateral_GC-ML-DG_ |  | -0.854 |  | 36 |  | 0.399 |  |
| bilateral_CA3_ |  | 0.503 |  | 36 |  | 0.618 |  |
| bilateral_CA4_ |  | -0.363 |  | 36 |  | 0.719 |  |
|  | | | | | | | |
| *Note.*  Student's t-test. | | | | | | | |
